# Molecular insights into titin’s A-band

**DOI:** 10.1007/s10974-023-09649-1

**Published:** 2023-05-31

**Authors:** Jennifer R. Fleming, Iljas Müller, Thomas Zacharchenko, Kay Diederichs, Olga Mayans

**Affiliations:** 1 Department of Biology, University of Konstanz, 78457 Konstanz, Germany; 2 Institute of Integrative Biology, University of Liverpool, Liverpool, L69 7ZB UK; 3 Wellcome Centre for Cell-Matrix Research, Manchester Academic Health Science Centre, University of Manchester, Manchester, UK

**Keywords:** poly-FnIII tandem, Pairwise sequence similarity, Sequence conservation 3D-mapping, X-ray crystallography, Titin evolution

## Abstract

The thick filament-associated A-band region of titin is a highly repetitive component of the titin chain with important scaffolding properties that support thick filament assembly. It also has a demonstrated link to human disease. Despite its functional significance, it remains a largely uncharacterized part of the titin protein. Here, we have performed an analysis of sequence and structure conservation of A-band titin, with emphasis on poly-FnIII tandem components. Specifically, we have applied multi-dimensional sequence pairwise similarity analysis to FnIII domains and complemented this with the crystallographic elucidation of the 3D-structure of the FnIII-triplet A84-A86 from the fourth long super-repeat in the C-zone (C4). Structural models serve here as templates to map sequence conservation onto super-repeat C4, which we show is a prototypical representative of titin’s C-zone. This templating identifies positionally conserved residue clusters in C super-repeats with the potential of mediating interactions to thick-filament components. Conservation localizes to two super-repeat positions: Ig domains in position 1 and FnIII domains in position 7. The analysis also allows conclusions to be drawn on the conserved architecture of titin’s A-band, as well as revisiting and expanding the evolutionary model of titin’s A-band.

**Supplementary Information:**

The online version contains supplementary material available at 10.1007/s10974-023-09649-1.

## Introduction

Titin is an intrasarcomeric protein filament of vertebrate striated muscle that plays essential roles in sarcomere development, passive mechanics and homeostatic regulation (reviewed in Linke 2018; Loescher et al., 2022). The titin polypeptide chain is composed of > 33,000 amino acids and extends from the Z-disc to the M-line, spanning the various functional regions of the sarcomere. Despite its large size, titin has a simple modular structure, primarily consisting of small immunoglobulin (Ig) and fibronectin type III (FnIII) domains (ca. 100 residues in length) that account for over 90% of its mass (Bang et al. 2001). Ig and FnIII domains are joined by short linker sequences and form poly-domain tandems that are interspersed by unique sequences in the titin chain - the PEVK-rich region in the I-band being a prominent example among the latter. Tandems composed exclusively of Ig domains are found in the elastic I-band region of titin (Bang et al. 2001). They are dynamically extensible and function as weak molecular springs, contributing to the passive elasticity of titin (Linke and Granzier 1998; Linke et al. 1999). Tandems composed of FnIII and Ig domains are found in titin’s A-band that is an integral component of the thick filament (Bang et al. 2001). There, they play a structural role, acting as stabilizing scaffolds that support interactions with thick-filament components (Bennett et al. 2020 and references within).

**Fig. 1:**
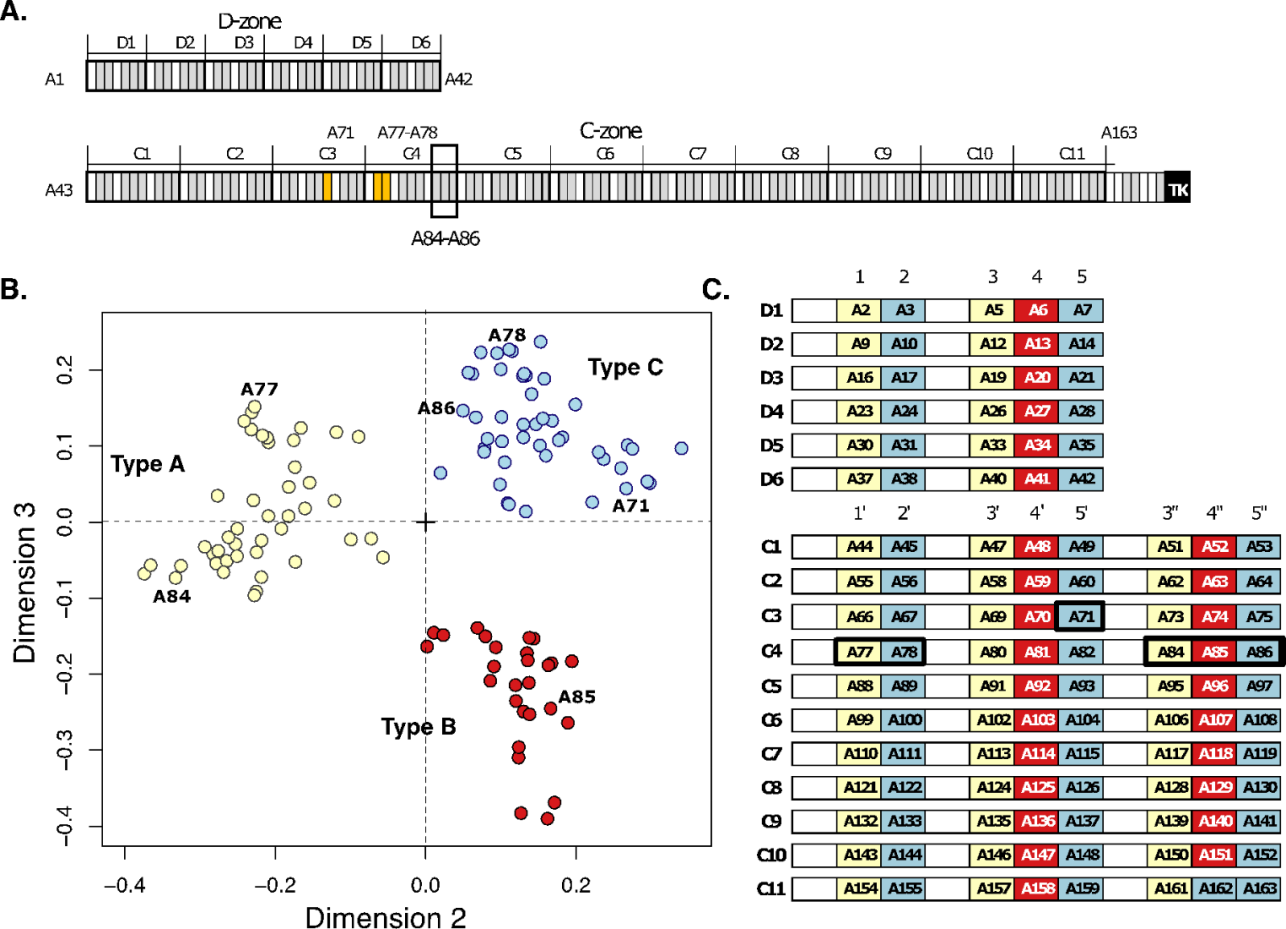
**PaSiMap analysis of FnIII-domain sequences from the D- and C- zone of titin**. **(A)** Schematic representation of the organization of domains into super-repeats in the D- and C-zones of A-band titin. FnIII modules are shown in grey and Ig domains in white; as reference the titin kinase domain in the sarcomeric M-line is displayed in black. Super-repeat boundaries are indicated by dividing lines. FnIII domains with previously known atomic structure are shown in yellow. The A84-A86 tandem structurally characterized in this study is boxed; **(B)** 2D-vector map of pairwise similarities for 117 FnIII domain sequences generated using PaSiMap (Su et al. 2022). Coordinates are colour-coded according to angular spread revealing the existence of three clusters corresponding to three FnIII sequence types. Domains for which 3D-structures are available are labelled; **(C)** Representation of tandem units of the D- and C-zone, where individual FnIII components are labelled and coloured according to cluster membership in B. Domains for which crystal structures are available are highlighted by a black box. Ig domains are unlabelled and shown as white boxes. (Positional numbering of FnIII domains within the tandem is as in Bucher et al. 2010)

In titin, groups of domains are repeated along the chain, with the pattern and composition of the repeat units reflecting the evolution of the protein through serial genetic duplication events. Tandem repeats in titin have been identified in the poly-Ig region of its I-band (Higgins et al. 1994; Kenny et al. 1999; Su et al. 2022), in the intrinsically disordered PEVK-rich sequence also in the I-band (Greaser 2001) and, most particularly, in the thick-filament associated A-band segment (Labeit et al. 1992; Higgins et al. 1994; Kenny et al. 1999). While I-band repeats are derogated and weakly distinguishable, it is the domain repeats in the A-band that are most distinct, showing a pronounced arrangement into super-repeats (i.e. repeats of repeats). Specifically, two zones are found in titin’s A-band: a D(distal)-zone and a C(central zone). The D-zone is found at the edge of the A-band, colocalizing with the tip of the myosin filament, and is formed by six copies of a short 7-domain tandem unit of composition [Ig-(FnIII)_2_-Ig-(FnIII)_3_]. The C(central)-zone extends along the myosin filament up to the edge of the bare zone and is formed by 11 copies of a long 11-domain tandem unit consisting of [Ig-(FnIII)_2_-Ig-(FnIII)_3_-Ig-(FnIII)_3_] (Fig. 1A). While various poly-Ig tandems from titin’s I-band have been characterized structurally (von Castelmur et al. 2008; Bogomolovas et al. 2016; Stronczek et al. 2021; Stergiou et al. 2023) and their interdomain dynamics studied through simulations at the atomic level (Lee et al. 2010; Bogomolovas et al. 2016) leading to a good understanding of titin’s I-band, the A-band super-repeats remain largely unstudied, with neither their structure nor the interactions they form with thick filament components being known at present. Currently, a single crystal structure is available that illustrates a FnIII-FnIII tandem arrangement from titin’s A-band, that of the duplex A77-A78 (Bucher et al. 2010). This is complemented by the early structure of the isolated FnIII A71 elucidated using NMR spectroscopy (Goll et al. 1998). No other 3D-structures of A-band components are currently available, although low-resolution small angle X-ray scattering data on A59-A60, A60-A62, A67-A68 (Tskhovrebova et al. 2010) and A77-A78, A80-A82 and A84-A86 (Bucher et al. 2010) have provided insights into tandem conformation and dynamics. Thus, at present, the scarcity of molecular data on titin’s A-band is notable.

The super-repeat composition of titin’s A-band is thought to assist thick filament assembly by acting as a template that provides regularly spaced anchoring sites for filament components, particularly myosin and myosin-binding protein C (MyBP-C). This view is supported by the co-localization of titin, myosin and MyBP-C in muscle micrographs (e.g. Tonino et al. 2019; Bennett et al. 2020) and the observation of their interaction in vitro (e.g. Labeit et al. 1992; Soteriou et al. 1993; Houmeida et al. 1995; Freiburg and Gautel 1996). Specifically, titin’s A-band structure correlates closely with that of the thick filament. The length of the long 11-domain repeat unit of C-zone titin, ~ 43 nm, coincides with the helical pitch of the myosin filament (Huxley 1963, 1967) and with the regular spacing of MyBP-C (Tonino et al. 2019; Bennett et al. 2020, and references within). MyBP-C lies transversal to the thick filament axis, forming 9 regularly-spaced stripes (numbered from the sarcomeric M-line) at intervals of ~ 43 nm and mapping to the interface between titin C-zone super-repeats, starting in C2-C3 until C10-C11 (but not C1-C2 or the interface between the end of the D-zone and C1) (Tonino et al. 2019; Bennett et al. 2020). By means of its interactions, titin has been proposed to act as a “molecular ruler” that determines the precise assembly length and exact subunit composition of the thick filament in the sarcomere (e.g. Whiting et al. 1989; Tskhovrebova and Trinick 2017). In brief, this hypothesis proposes that an interaction between titin (the ruler) and myosin (the growing polymer) along the A-band determines the conserved length of the myosin polymer in the sarcomere, which is ∼1.6 μm in many vertebrates and involves a consistent number of subunits (294 subunits) (Huxley 1963, 1967). A change in polymerization conditions can be expected to occur when the myosin polymer reaches the end of the ruler, in this case titin’s I/A junction that has a different domain composition and, thereby, stops supporting polymer growth (Bennett and Gautel 1996). Unexpectedly, a mouse model in which titin’s I/A junction had been deleted did not reveal an altered thick filament length (Granzier et al. 2014). However, support to the “molecular ruler” model was finally brought by a mouse model in which two of titin’s C-zone super-repeats were deleted, resulting in a shortening of the thick filament length by the expected amount (Tonino et al. 2017).

To date, the molecular characterization of interactions between titin, myosin and MyBP-C is largely lacking. The binding of titin to myosin appears to involve primarily the light meromyosin (LMM) region (Labeit et al. 1992; Soteriou et al. 1993; Houmeida et al. 1995), although short recombinant FnIII tandems were shown to interact weakly with the myosin head (Muhle-Goll et al. 2001). The interaction of titin and MyBP-C is of special interest as MyBP-C plays an important role in modulating the acto-myosin interaction during muscle contraction and its mutation is critically associated with heart disease, being responsible for ~ 40% of all known mutations leading to hypertrophic cardiomyopathy (Heling et al. 2020). MyBP-C consists of 10–11 Ig and FnIII domains (according to isoform). Its C-terminal domains bind titin as well as the myosin tail and run axially along the thick filament towards the M-line, while the N-terminal protein fraction is transversally oriented in the sarcomere, interacting with both myosin heads and actin (recently reviewed in Heling et al. 2020). Using recombinant samples, the MyBP-C binding site in titin was shown to map to the first Ig domain of each long super-repeat in the C-zone (Freiburg and Gautel 1996). However, the fact that the three C-terminal domains of cMyBP-C run along the thick filament (Lee et al. 2015) leads to the expectation that additional titin domains are involved in the interaction. As the binding localizes to titin super-repeat interfaces, it is hypothesized that the terminal FnIII domains in each preceding super-repeat are also involved (Tonino et al. 2019). In brief, the exact mapping of binding sites mediating the myosin/MyPB-C/titin interaction in the thick filament remains a pending and challenging task.

In agreement with its structural and developmental role in the sarcomere, titin’s A-band is of high biomedical significance, with many disease-causing mutations localizing to this region of the chain. In particular, titin truncating mutations often occur in the A-band (Herman et al. 2012) and are a leading cause of dilated cardiomyopathy (DCM) (Roberts et al. 2015), causing 25% of familial cases of idiopathic dilated cardiomyopathy and 18% of sporadic cases (Herman et al. 2012). Recently, the truncation of the A/I junction in titin has also been linked to DCM (Akinrinade et al. 2016). However, titin truncating A-band variants can have a broad phenotypic spectrum and they have also been linked to various skeletal myopathies (Rich et al. 2020). In addition to truncating mutations, many missense variants are also found in titin’s A-band, but their pathological significance remains difficult to establish. Interestingly, missense mutations linked to cardiomyopathies have been found preferentially in the three terminal FnIII domains of C-zone super-repeats, but no unique role of these FnIII domains has been identified that provides an explanation (Begay et al. 2015). It has been proposed that missense variants can lead to local domain unfolding, phenocopy truncation effects and/or have a modifier effect on other mutations (Rees et al. 2021). Understanding the structure and interactions of titin within the thick-filament will contribute to rationalize its role in disease and will aid future studies on disease linkage.

In order to advance the molecular understanding of titin’s A-band, we examine here sequence relationships across FnIII domains in A-band tandems by applying multi-dimensional cluster-analysis in PaSiMap, which has been applied recently to the re-examination of Ig components from titin (Su et al. 2022). PaSiMap sensitivity allowed for the identification of Ig sequences from titin that had diversified from the main groups and revealed the existence of a previously unidentified domain repeat in the distal I-band (Su et al. 2022). Here, we perform PaSiMap analysis on A-band FnIII domains to reveal a fine gradient of conservation in the tandem repeats and identify prototypical representative components of this region of titin. We complement this analysis with the crystallographic elucidation of the triple-FnIII tandem A84-A86 from super-repeat C4, which illustrates the structural properties of equivalent C-zone tandems. Finally, 3D-structures are used as templates to interpret conservation patterns across A-band modules and propose candidate residues forming binding interfaces to protein components of the thick filament.

## Methods

### FnIII-domain protein sequences

117 sequences of FnIII domains forming the C- and D-zones of titin’s A-band were obtained by translation of the inferred complete titin metatranscript (NCBI NM_001267550.2; UniProtKb Q8WZ42). Sequence borders for each domain were defined as follows: N-termini as given in (Bucher et al. 2010); C-termini as deduced from structural alignments of crystal structures of A77, A78 (both domains contained in PDB entry 3LPW), A84, A85 and A86 (in this work). Thus, the domain definition excluded linker sequences, which were not part of the analysis.

Throughout this study, we use the domain numbering nomenclature from Bucher et al. 2010 (given in Fig. 1B) that reflects both the positional and evolutionary relationships of the FnIII tandems. FnIII domains in the D-zone are numbered 1–5, then those in the C-zone are numbered 1’ to 5’ with the last three domains numbered 3”-5”.

### PaSiMaP sequence comparison

Comparison of FnIII sequences was performed using PaSiMap (Su et al. 2022) executed through its web server (http://pasimap.biologie.uni-konstanz.de). To graphically visualize sequence similarities, 3D- and 2D-representations of the resulting PaSiMap vector maps were made in R (https://www.R-project.org). Eigenvalues showed that the variability in the sequence set was well accounted for by the first three dimensions. These were then used in the 3D-visualization of the resulting groups (Supp Materials Data). To ease graphical presentation in this manuscript, the sequences were mapped next as coordinates in 2D space using the second and third dimensions (D2 and D3), which reveal the finer detail of the clustering partition. To investigate higher dimensions (i.e. sub-groupings of finer detail), individual clusters identified in the initial PaSiMap study were subjected to a further round of PaSiMap analysis.

### Protein production

The expression clone for A84-A86 from human titin (residues 23,562 − 23,866; EMBL access code X90568) has been previously described (Muhle-Goll et al. 2001).

Protein expression was in *Escherichia coli* strain BL21 (DE3) Rosetta grown at 37 °C up to an OD_600_ of 0.6 in Luria-Bertani medium supplemented with 25 µg/ml kanamycin and 34 µg/ml chloramphenicol. Expression was induced with 1 mM isopropyl-β-D-thiogalactopyranoside (IPTG) and growth continued for 16 h at 30 °C. Bacterial pellets, harvested by centrifugation at 3400 *g* and 4 °C, were resuspended in lysis buffer composed of 100 mM NaCl, 50 mM Tris-HCl pH 7.2, 2 mM DTT in the presence of a protease inhibitor cocktail (Boehringer) and DNAse. Lysis was performed by sonication. The homogenate was clarified by centrifugation and applied to a Ni^2+^-chelating HisTrap column (Cytiva) equilibrated in lysis buffer. Elution was performed with 200 mM imidazole. The eluate was dialyzed against 150 mM NaCl, 50 mM Tris-HCl pH 8.0, 2 mM DTT in the presence of Tobacco Etch Virus (TEV) protease at 4 °C overnight for tag removal. Subsequent purification used reverse metal-affinity chromatography on a HisTrap column, followed by gel filtration on a Superdex 75 HiLoad 16/60 column (both from Cytiva) in lysis buffer. Resulting samples were stored at 4 °C until further use.

### Crystal structure elucidation

Crystals of A84-A86 were grown on VDX crystallization plates at 4 °C using the hanging-drop method. Drops consisted of 1.5 µl of protein solution at 40 mg/ml and 0.5 µl of polyethylene glycol 400 at a final concentration of 5% [v/v]. Reservoir solutions contained 5% [v/v] polyethylene glycol 400 and 40% [v/v] 2-methyl-2,4-pentanediol (MPD).

X-ray diffraction data were collected at the DIAMOND synchrotron (Didcot, UK) on beamline I04 under cryo-conditions (100 K) from crystals that had been vitrified in liquid nitrogen directly in native mother liquor. Data processing used the XDS suite (Kabsch 2010). Phasing was performed by molecular replacement in PHASER (McCoy et al. 2007) using the crystal structure of fibronectin domain A170 from titin (extracted from PDB entry 2NZI; Mrosek et al. 2007) as search model. A170 accounts for one-sixth of the asymmetric unit content of this crystal form, which consists of two molecular copies of A84-A86 related by non-crystallographic symmetry. Manual model building was performed in COOT (Emsley et al. 2010) and refinement in Phenix.refine (Liebschner et al. 2019) using non-crystallographic symmetry (NCS) restraints, isotropic B-factors and TLS refinement (each individual FnIII domain was declared as a NCS and TLS group). Statistics for X-ray data processing and model refinement are given in Table 1.

**Table 1:** X-ray diffraction data and model refinement statistics

	A84-A86
PDB code	8BNQ
Space group	*P*1
Cell dimensions:	
a, b, c (Å) α, β, γ (°)	31.269, 75.003, 79.839 114.574, 92.503, 100.913
Copies in ASU	2
**Data Processing**	
Beamline	I04 (Diamond)
Detector	PILATUS 6 M
Wavelength (Å)	0.97250
Resolution (Å)	29.50–2.30 (2.35–2.30)^a^
No. Reflections	27,975 (2819)
R_meas_(I) (%)	12.1 (162.1)
<I/σI>	9.27 (1.02)
CC1/2 (%)	99.6 (49.2)
Completeness (%)	97.95 (97.10)
Multiplicity	3.6 (3.6)
**Model Refinement**	
No. working/free reflections	27,954 (1001)
Rwork/Rfree (%)	19.7/25.5
No. Protein residues	607
No. Atoms Solvent	132 H_2_O; 2 x EGO^b^
R.m.s.d. bond length (Å)	0.009
R.m.s.d. angles (°)	1.103
Ramachandran plot	
Favoured/disallowed (%)	95.52/0.17
Number of TLS groups	6

^a^ values in parenthesis are those of the highest resolution shell.

^b^ EGO, Ethylene glycol.

### Identification of a sequence consensus across FnIII domains from titin’s A-band

In order to define a global conservation consensus for all 117 FnIII sequences, we identified the sequence features deterministic of the original PaSiMap output by implementing a differential PaSiMap analysis as follows. First, a multiple sequence alignment (MSA) was calculated for the 117 FnIII sequences with Clustal Omega using default settings (https://www.ebi.ac.uk/Tools/msa/clustalo). Then, in the resulting global MSA, the conservation of each residue position was calculated by means of a Sum-of-Pairs-Score (SoP Score) using the BLOSUM62 matrix and unweighted sequences as described in AL2CO (Pei and Grishin 2001). In brief, the SoP Score is calculated per position by multiplying the BLOSUM62 score of each pair of residues found in that position by the probability of drawing that pair. Then all pair scores for that MSA column are added together to issue a resulting SoP Score for that position of the MSA. To avoid overestimating the conservation of minimally occupied positions (i.e. positions occupied in some sequences but being a gap in others), the SoP score was set to 0 for positions with a threshold ≥ 50% gaps. The threshold of 50% gaps was chosen to avoid the effect of spurious high conservation scores caused by sub-alignments in under-occupied positions and is the threshold suggested in AL2CO (Pei and Grishin 2001). The global MSA alignment and the SoP score for each residue position is provided as Supp Fig S1.

Next, to define the SoP threshold that makes a difference to PaSiMap output the following procedure was used: residue positions in the global MSA were ranked according to their conservation (i.e. SoP Score) from higher to lower, then the impact of each position on the PaSiMap output was evaluated. For this, a series of ‘derivative MSAs’ were created by deleting each position cumulatively by order of conservation (i.e. highest SoP score first) and replacing this position with a gap. For each of these derivative MSAs, PaSiMap was applied (without prior pairwise sequence alignment - i.e. lines from the MSA were compared as-is to obtain PaSiMap pairwise similarity scores). The PaSiMap vector map obtained for each derivative MSA was then compared with the original PaSiMap map calculated from the unedited original MSA. This comparison used a modified version of the program *cc_analysis.compare_solutions*, which calculates the root-mean-square deviation (RMSD) values between vectors (Kabsch 1976, 1978). Thereby, the RMSD value between two PaSiMap vector maps quantifies the effect of removing one or more residue positions on the PaSiMap output; the higher the RMSD value, the greater the effect. To correct for the bias of removing positions from an increasingly small pool of residues (in other words, a change that represents the removal of one out of 10 positions will have a greater influence than removing one out of 30) a weighted RMSD score was used that was calculated as follows:

1 $$\Delta RMSD(i) = RMSD(i) - RMSD(i - 1)$$

2 $$w(i) = \frac{{{i_{\max }} - i}}{{{i_{\max }}}}$$

3 $$adjusted\,RMSD(i) = \sum\limits_{n = 0}^i {w(n)\,\Delta RMSD(n)}$$

*i* = number of removed positions.

*i*_max_ was chosen so that the last position removed still had a conservation score > 0.

To establish a comparative baseline for the effect of removing residues randomly, a sham derivative MSA was tested for each cumulative removal stage, where the equivalent number of positions were removed from the MSA at random. In each case, three ‘random derivative MSA’ were calculated and the RMSD values obtained averaged. The difference in RMSD values obtained from comparing the original PaSiMap vector map with its true derivatives and with the corresponding sham derivatives guided the determination of the SoP threshold defining conservation in the MSA. Namely, conserved residue positions were defined here as those whose removal resulted in a positive slope in the curve of differential, weighted RMSD values. While residue positions whose difference in RMSD values led to null or decreased slopes, were taken as non-defining of PaSiMap output Supp Fig S2. This approach led to defining the SoP score of conservation as 0.5, so that all residue positions with a higher SoP score value were regarded as conserved and, thereby, as constituting the conservation consensus for FnIII domains from titin’s A-band.

### Calculation of sequence conservation specific to each super-repeat position

In order to identify candidate residues potentially forming a binding interface to proteins within the thick filament, we aimed to determine the sequence conservation specific to each domain position within the C-zone super-repeat. For this, we removed from sequences (i) conserved residues identified in the global MSA (as described above) and thus shared by all A-band FnIII; and (ii) conserved residues for each FnIII group (i.e. FnIII-type A, B and C) corresponding to clusters in the original PaSiMaP vector map. For the latter (ii), an FnIII-type specific MSA was generated containing FnIII sequences for domains in each group but where the globally conserved residue positions previously identified in the global MSA had been removed and substituted with gaps, and a new SoP conservation threshold defined using the procedure described above for the global MSA. Here, to ensure an equal selection of residues across groups, a cut-off SoP of 1 was used for all groups. (FnIII type-specific MSA alignments and the corresponding residues identified in this way are shown in Supp Fig S3). Next, sequence subgroups were generated that consisted of domains at equivalent repeat positions across the C-zone super-repeats C2-C11. D-zone components as well as domains within repeat C1 were discarded, as they are known not to support the same interactions with thick filament components. For each position-specific MSA (where globally conserved and FnIII-type conserved residues had been removed and substituted by gaps), the remaining residues were re-assessed for conservation using the following criteria to define conservation: SoP > = 1, at least 90% occupancy of the residue position in the alignment and amino acid identity of at least 70% where the following identity groups were used (TIL, DEN, FY) – residue types not specified were not grouped. A conservative, higher SoP score of 1 was chosen here to account for the smaller number of sequences and as this is the smallest positive score in the BLOSUM62 matrix, corresponding to the smallest conservation possible for similar residues.

## Results

### FnIII domain relationships in titin’s A-band revealed by cluster analysis in PaSiMaP

In order to analyse sequence similarity relationships across FnIII domains from the A-band of titin, 117 domain sequences were subjected to an analysis of pairwise similarities mapped spatially using PaSiMap (Su et al. 2022). PaSiMap is an algorithm that allows for the classification of sequences based on their systematic differences to other sequences within a set. It represents protein sequences as coordinates in multi-dimensional space based on their pairwise similarities, where each coordinate constitutes a vector from the origin. The vector angle reflects the systematic differences between sequences, where two given sequences with a similar angle will have shared features but be systematically different to other sequences with a different vector angle. Sequences that differ systematically do so in their distinct features, so that a group of sequences sharing the same defining features will form a spatial cluster in PaSiMap maps. The vector length from the origin reflects how strong the signal is with respect to other sequences with a similar angle, i.e. how dominant the systematic features are in the sequence. PaSiMap separates the sequences over many dimensions, with each dimension representing a unique systematic difference between the sequence groups. Similar to principal component analysis (PCA), also in PaSiMap dimensions are hierarchically ordered by the level in which they describe features of the dataset. The first dimension (1D) represents the most influential feature of the sequences, the second dimension (2D) maps a finer orthogonal feature, and so forth.

PaSiMap analysis revealed that differences between sequences are well accounted for by three dimensions, as shown by the examination of eigenvalues. The 3D-vector map resulting from PaSiMap in this study, where individual domains can be identified interactively by point-and-click, is provided as Supp Materials Data. 2D-maps corresponding to the second (D2) and third (D3) dimensions, which correspond to finer differences in the sequences and thereby offer higher mapping resolution and better cluster visualization, are shown here (Fig. 1B). The map revealed a segregation of FnIII domain sequences into three distinct coordinate clusters, corresponding to three identifiable FnIII sequence types, here termed types A, B and C. When FnIII type (according to cluster membership) was mapped onto the domain components of D- and C-zone repeats, a clear pattern emerged (Fig. 1C). Here, FnIII type A is found always in N-terminal position in any given FnIII-tandem (whether dual or triple FnIII composition), i.e. in positions 1 and 3 in the D-zone repeats and positions 1’, 3’ and 3’’ of the longer C-zone repeats; FnIII type C is always located in C-terminal position in any given FnIII-tandem, i.e. in positions 2 and 5 in D-zone repeats and positions 2’, 5’ and 5’’ of the C-zone repeats; while FnIII type B is found in central position in the triple-tandems of the D- and C-zone and, thereby, not present in tandems composed of only two FnIII domains. The only exception to this pattern is domain A162, which is in a middle position of the last triple tandem of the C-zone, but belongs to type C instead of type B. This divergent triplet marks the end of the A-band segment of titin. Thus, the data reveal that the D-zone is formed by -AC-ABC repeat units and the C-zone by -AC-ABC-ABC units (where “-” corresponds to Ig domains). This agrees with an early evolutionary model that deduced that the insertion of the central domain in the triple FnIII-tandem occurred prior to the duplication of FnIII-tandems into the formation of D-zone and C-zone super-repeats and that the C-zone repeat unit evolved from the D-zone repeat unit by duplication of the triple tandem (Higgins et al. 1994; Kenny et al. 1999). Interestingly, PaSiMap results indicate that FnIII type B is equally distant from types A and C. Hence, its evolution from either of these types or an exogenous FnIII cannot be deduced from this sequence analysis alone.

### Identification of prototypical FnIII domains in titin’s A-band

**Fig. 2:**
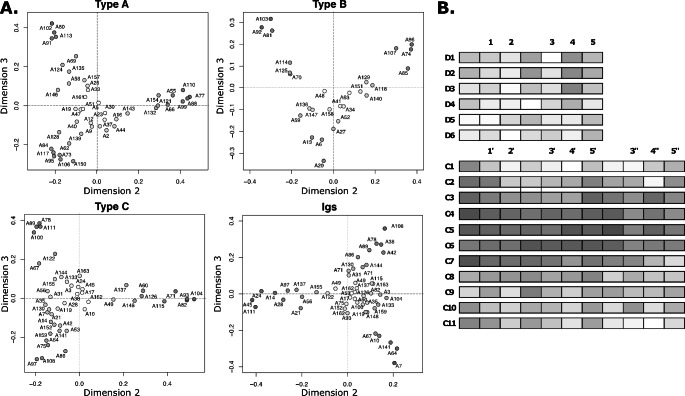
**Occurrence of distinct systematic differences in A-band domains**. **(A)** PaSiMap sub-analysis of individual FnIII groups as defined by clusters identified in Fig. 1B and A-band Ig mapping as defined in (Su et al. 2022). Sequence points are coloured in a white-to-grey gradient according to vector length from the origin, where dark grey (longest vector) corresponds to prototypic domains with dominant systematic differences and lighter grey (shorter vectors) corresponds to the most variable domains (white is the map origin: coordinates 0,0). **(B)** D- and C-zone super-repeats where individual domains are coloured as in A. (The degree of shading is relative to each vector map and, thus, can only be quantitatively compared within the same sequence group)

In order to identify finer differences within the identified FnIII types, we subjected each cluster of sequences to an individual reanalysis in PaSiMap. Coordinates in the resulting maps (Fig. 2A-C) were then shaded in a white-to-grey gradient according to their distance from the origin, where white corresponded to the origin and the darkest grey was assigned to the longest vector in each of the individual maps. Thus, dark grey indicated sequences with strongly defining systematic differences, while light shadowed coordinates indicated more diversified sequences with less dominant characteristic features. Consequently, this gradient allows for the identification of domains with the most prototypical features within a respective group. For completeness, we extended this analysis to Ig domain components of the super-repeats by building on recently reported PaSiMap vector maps of these domains (Su et al. 2022). PaSiMap results so shaded were then mapped onto domain components of the A-band repeats (Fig. 2D). This revealed that the most prototypical domains, both Ig and FnIII, are located within the central repeats of the C-zone, C4-C6. The pattern decreases progressively towards the termini of the C-zone, with the outer tandems C1-C2 and C9-C11 at the beginning and end of the C-zone, respectively, being the most diversified and presenting less prototypical features, jointly with the D-zone tandems. In agreement with our observations, a previous analysis that quantitated sequence conservation in multi-sequence alignments of titin’s A-band components (Amodeo et al. 2001), concluded that domains in super-repeats C3-C6 shared the highest similarity. It could be thus concluded that the display of strong defining features occurs at the tandem level, i.e. being common to all domains within the tandem, and not to specific tandem positions. The findings also suggest that genetic duplication occurred at the super-repeat level. Since a functional constraint that preferentially slowed the mutational rate of these central tandems is not known, it might be deduced that the central repeats have emerged more recently, in agreement with a previous proposal (Amodeo et al. 2001). Moreover, the gradient of conservation now revealed suggests that the C-zone has grown progressively, becoming gradually extended in its inner, middle part.

**Fig. 3:**
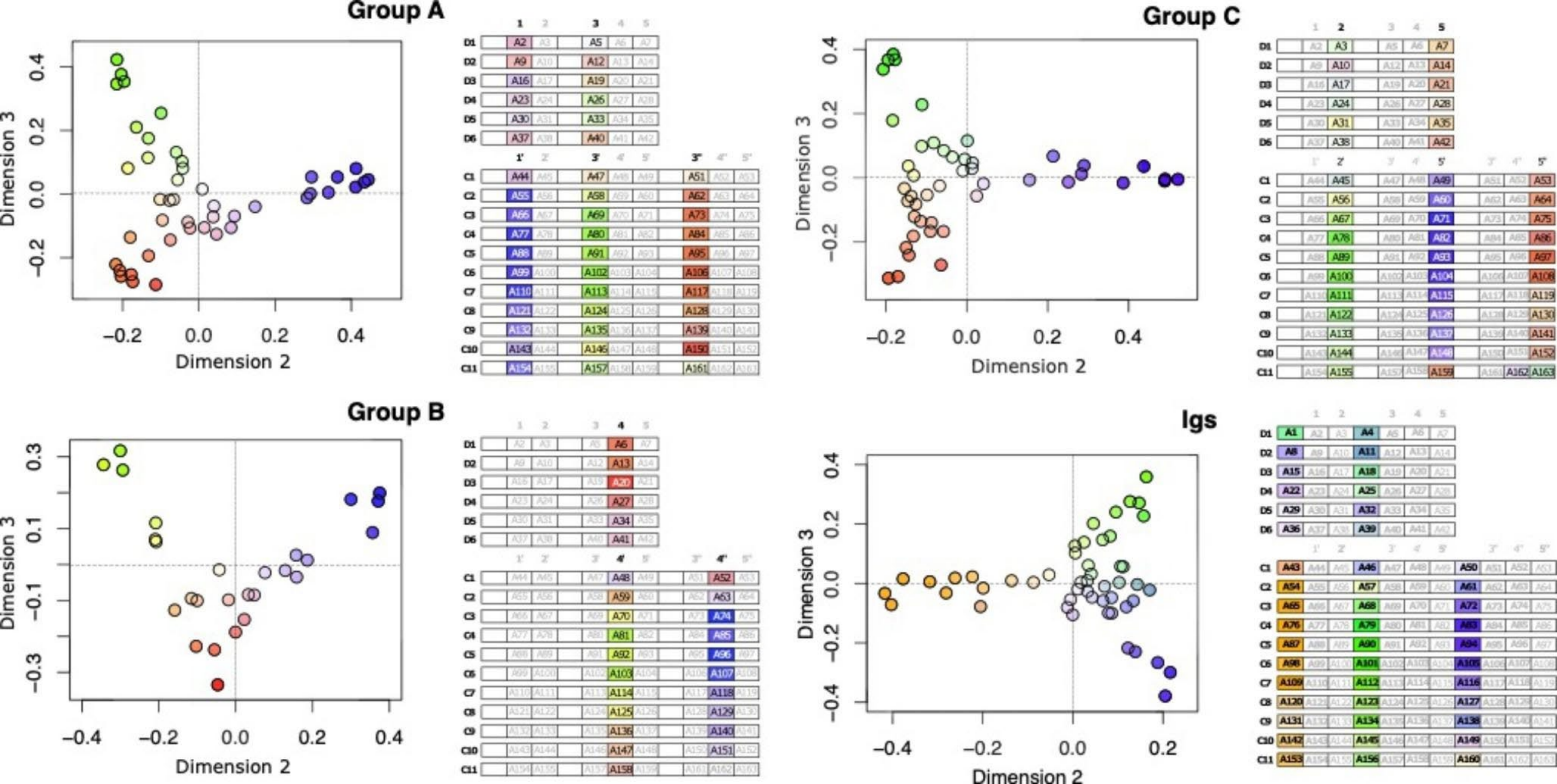
**Systematic sequence features correlate with domain position within the tandem**. PaSiMap sub-analysis of individual FnIII types (**A**, **B** and **C**) and Ig components of titin’s A-band. In vector maps, sequence coordinates are coloured according to angle using a rainbow spectrum and shading white-to-colour from the origin, with coordinates further from the origin being more colour saturated (white indicates the map origin; coordinates 0,0). For each vector map, corresponding D- and C-zone super-repeats are shown where individual domains are coloured as in maps. (Note: the colour gradient is calculated independently for each domain type and, thus, implies no relation across different types)

While vector length in PaSiMap maps indicates the dominance of distinct sequence features, it is the vector angle that correlates with the specific type of sequence features. To reveal the distribution of shared sequence features across FnIII domains, in addition to shading, coordinates were coloured by their angle in 2D-maps applying a rainbow gradient (Fig. 3; left). The mapping of this colour scheme onto super-repeats (Fig. 3; right) revealed a further pattern that correlates with the position of domains within the tandem and in agreement with a model where A-band titin has evolved through recursive duplications of super-repeats. This agrees with the earlier observation that domains at comparable positions in C-zone super-repeats are more similar to each other than to others within the repeat (Amodeo et al. 2001). We found, however, that this effect is pronounced only in the central super-repeats of the C-zone. The effect is minor in the first and last repeats C1 and C11 and it is barely observable in the D-zone, where only position 4 (i.e. FnIII domains of type B in central position in the triple tandems) shows distinct systematic features, potentially reflecting the later evolution of this domain within the tandem.

### The crystal structure of the A84-A86 tandem from C-zone repeat 4 (C4)

PaSiMap analysis suggested that FnIII domains from A-band titin occur in three different types: A, B, C. We asked whether the sequence differences across types translated into structural differences in the corresponding domains or in their arraying mode within the titin chain. To date, the A-band of titin remains largely uncharacterized at the molecular level, with experimental 3D-structures only available for the A77-A78 duet from the C4 super-repeat (Bucher et al. 2010) and the isolated A71 from C3 (Goll et al. 1998) (Fig. 1A). Of these, only the crystal structure of A77-A78 shows domains in tandem, being so far the only observation of FnIII domain arrangements in titin’s A-band. Domain A77 belongs to group A identified in this study, while domains A78 and A71 are of type C (Fig. 1C). In order to obtain a domain structure representative of group B and to expand the understanding of FnIII domain arraying in titin’s A-band, we sought to elucidate the crystal structures of the triple FnIII-tandems A80-A82 and A84-A86 (both tandems of type ABC) from the C4 super-repeat, which PaSiMap analysis reveals to be a prototypical representative of titin’s C-zone (Fig. 2D). We succeeded in solving the 3D-structure of A84-A86 using X-ray crystallography, while no crystals could be obtained from A80-A82.

**Fig. 4:**
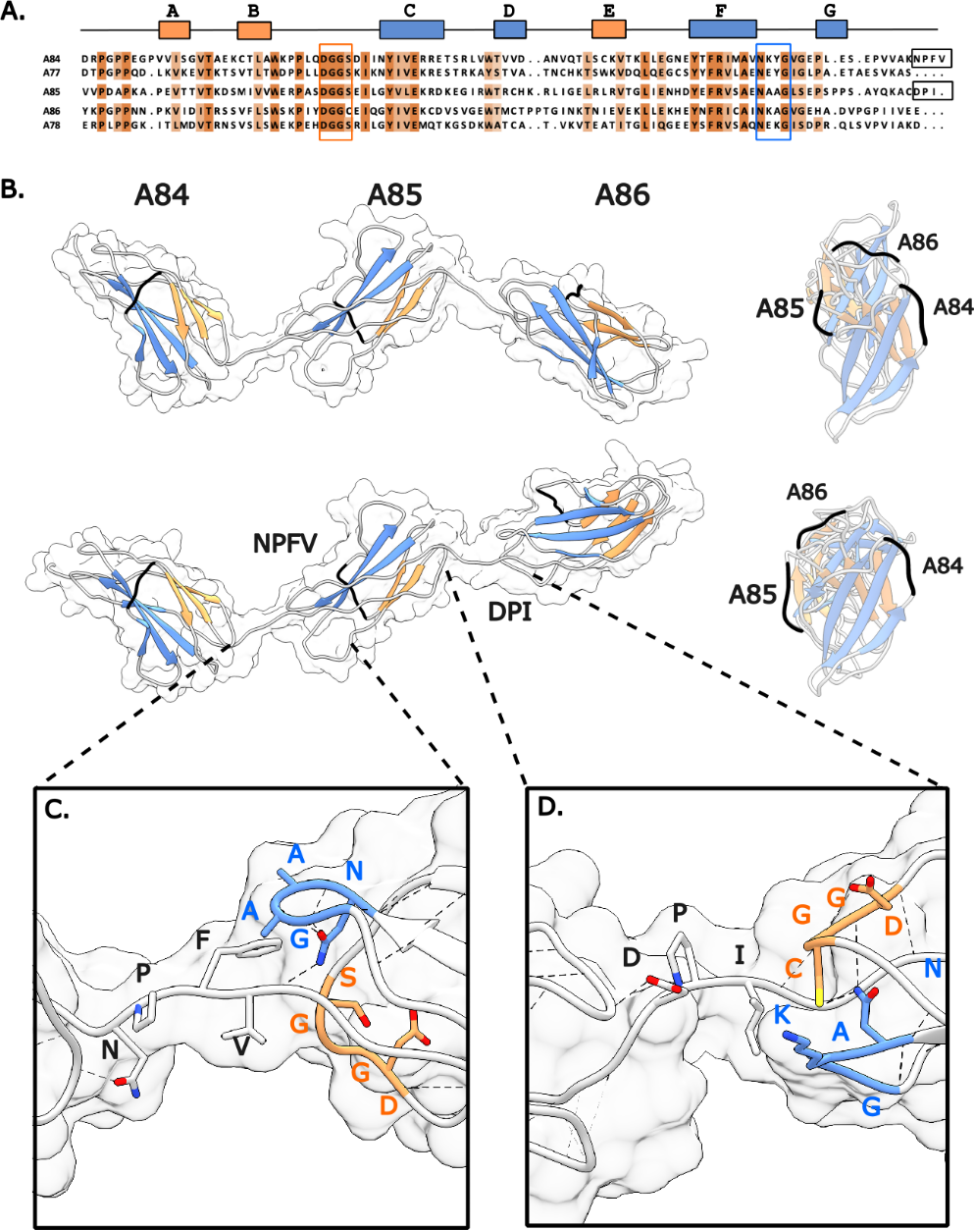
**Crystal structure of A84-A86 in two extended conformations**. **(A)** Sequence alignment of C-zone tandems of known crystal structure. β-strands are indicated as boxes above the sequence. Loops involved in interactions with linker sequences and, thus, part of domain-domain junctions are boxed in red and blue; **(B)** Crystal structure of A84-A86 in two extended conformations. The structures have been aligned on their central domain (A85) to better illustrate similarities and differences in the position of the remaining domains. β-sheets are coloured orange and blue (colour-coded as in A) and the DE loop is highlighted in black to ease the visualization of domain torsions. Lateral (left) and longitudinal (right) views are provided; **C** and **D.** Interdomain regions of A84-A85 and A85-A86, respectively. Hydrogen bonds are shown as dashed lines. Key residues in conserved loop motifs and constituting the interface between the linker and the subsequent domain are shown as sticks and colour-coded as in A. No direct contacts between domains are observed. (Equivalent interfaces for the remaining molecular copy in the crystallographic asymmetric unit are shown in Fig S7)

The crystal structure of A84-A86 has been elucidated to 2.30 Å resolution (data and model statistics are given in Table 1). The crystal form obtained in this study contained two molecular copies of A84-A86 in its asymmetric unit that corresponded to two related extended conformations of the tandem (described below). At the domain level, individual FnIII components were structurally equivalent in both molecular copies (RMSD_Cα_ values of 0.84 Å, 0.35 Å and 0.44 Å across copies of A84, A85 and A86 domains, respectively; calculated using UCSD Chimera, Pettersen et al. 2004). In A84-A86, domains display characteristics typical of the canonical FnIII fold of titin (Amodeo et al. 2001; Bucher et al. 2010); namely, among other signature motifs, they contain (i) a buried tryptophan residue in β-strand B that is involved in hydrophobic core packing; (ii) a tyrosine residue in β-strand F that forms the classical “tyrosine corner” (Hamill et al. 2000); and (iii) a cluster of three interacting, highly conserved motifs that define the N-terminal pole of the fold consisting of motif Px[P]P in β-strand A, a Pxx[D]GG[S/C] in the BC loop and a AxNxxG motif in β-hairpin FG (Fig. 4A; Fig S1). In the latter motif, the highly conserved asparagine residue acts as a central anchor that joins β-hairpin FG to the BC loop and linker sequence, forming a conserved network of interactions in the N-terminal pole of the FnIII fold. These elements are shared across FnIII from A-band titin (Fig S1) and also resemble closely equivalent features in the related Ig domains of titin (Marino et al. 2005) so that, at the domain level, titin displays a certain structural homogeneity.

PaSiMap analysis revealed that, in addition to globally conserved features, the FnIII domains also possess specific features that lead to their classification in distinct types (A, B and C). Thus, we asked whether sequence differences translated into specific structural features in the three FnIII types by using available crystal structures (type A: A77 and A84; type B: A85; type C: A78 and A86) in a comparative structural analysis. For this, we did a global comparison as well as identified a small set of sequence features specific to each FnIII type (Supp Fig S3) and inspected their structural context and interactions in crystal structures. However, this did not reveal either significant fold differences or domain arraying characteristics that could be attributed to the specific sequence features. Thus, we conclude that sequence differences underlying the PaSiMap clusters are well accommodated by the FnIII fold and did not translate into significantly distinct structural phenotypes. The features are likely to be evolutionary remnants or have unidentified functional or structural roles.

Regarding overall conformation, the crystal structure of A84-A86 reveals the poly-Fn tandem chain in an extended arrangement where sequential domains display alternating torsions along the chain path (Fig. 4B). Domain pairs A84-A85 and A85-A86 are joined by 3 residue long linkers that are largely hydrophobic in composition, having sequence NPF and DPI, respectively. Direct contacts between domains that could provide stability and rigidity to the tandem conformation are not observed, with steric hindrance between linker groups and neighbouring domains favouring extended arrangements (Fig. 4C-D & Fig S7). Accordingly, small positional differences affect domains within the two molecular copies in the asymmetric unit of this crystal form. The copies differ primarily in the opening and rotation angle of the A85-A86 pair, which varies moderately between copies. An analysis of phi and psi dihedral torsion angles of linker residues showed that domain repositioning is singly caused by a small rotation around the psi main chain angle of the proline residue (DPI) in the linker sequence joining both domains. Little conformational difference exists otherwise in the linker sequences, where the presence of prolines (both in trans conformation) and bulky aromatic side chains seem to prevent linkers from acting as free interdomain hinges.

### Conservation of FnIII tandem conformation in titin’s A-band

**Fig. 5:**
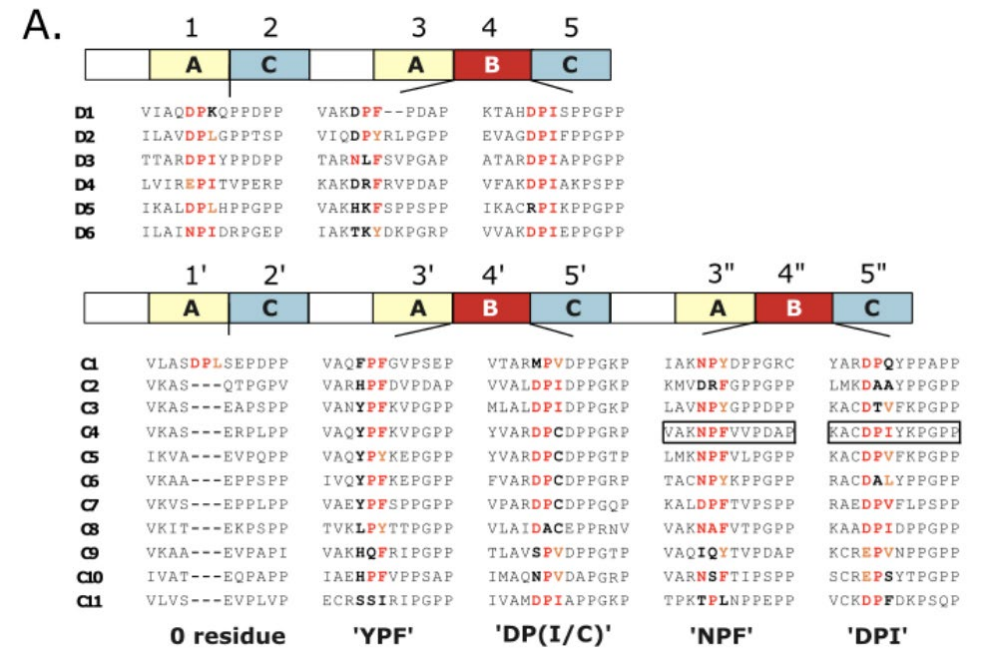
**Conservation of linker sequences in FnIII tandems of titin’s A-band**. Conservation in FnIII-interdomain linker sequences. Linker residues are indicated in bold. Residues identical to those in experimental structures are indicated in red. Close sequence conservation to residues in crystal structures is in orange

Conservation within titin’s A-band is not only detected at the domain level, but as a result of duplication occurring at the tandem level also includes interdomain features, specifically linker length and composition. We observed that three primary types of domain linkers are found between pairs of FnIII domains in D- and C-zone super-repeats: (i) ultra-short “zero-length” linkers joining positions 1’-2’ and 4’-5’, illustrated by the crystal structure of A77-A78 (Bucher et al. 2010); (ii) domains joined by a [N/Y]PF sequence as represented by the A84-A85 pair in this study; and (iii) domains linked by a [D]PI motif as A85-A86 also in this work (Fig. 5). The short “zero-length” linker results in domains that pack tightly along the titin chain, displaying numerous interdomain contacts and with a stiff domain-domain conformation (stiff conformation is to be understood here as that which does not allow interdomain motions; i.e. modular movements, if present, are small compared to the size of the domains) (Bucher et al. 2010; Zacharchenko et al. 2015). The [N/Y]PF sequence, with its bulky hydrophobic residue components, appears to allow only minor interdomain flexibility as revealed by a comparison of the two molecular copies of A84-A86 in this study. The [D]PI linker is more flexible (as described above). Zero-length, [N/Y]PF and [D]PI linkers (and close derivative sequences) are periodically distributed in A-band repeats (Fig. 5). A zero-length linker is found in C-zone (C2-C11) pairs 1’-2’; the [D]PI linker is found in D-zone pairs 1–2 and 4–5 as well as C-zone 4’-5’ and 4”-5”; the [N/Y]PF linker occurs in D-zone pairs 3–4 and C-zone pairs 3’-4’ and 3”-4”. An analysis of the degeneracy of this latter linker sequence, particularly in the D-zone, leads us to speculate that it derives from the [D]PI linker. The study of pairs 1’-2’ in the C-zone suggests the possibility of the zero-linker having evolved last as an *indel* by deletion of an original [D]PI linker, with only the first pair in the zone retaining the conserved, original linker. This suggests that the deletion of the linker must have occurred prior to the duplication of the C-zone super-repeats.

**Fig. 6:**
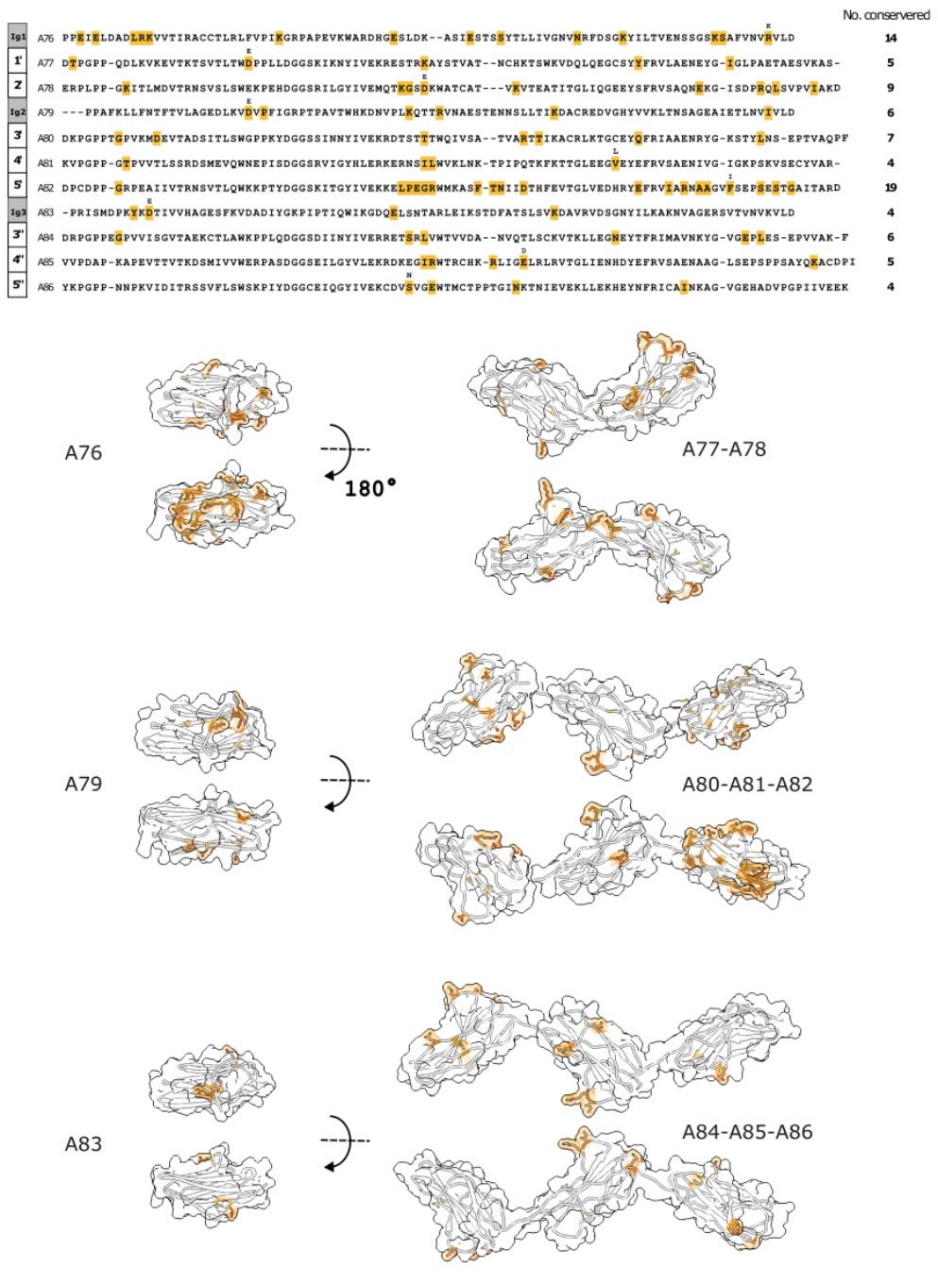
**Sequence conservation specific to each domain position within the C-zone super-repeat**. **Top**: Residues with highest conservation specific to domain position in the super-repeats C2-C11 are highlighted in orange on the sequences from components of the C4 tandem (A76-A86). Residues that differ from the reference sequence or have a mixed consensus are shown above the sequence; **Bottom**: Mapping of conserved residues onto structural models. Conserved residues are coloured orange (as above). Ig models were obtained from the AlphaFold database (https://alphafold.ebi.ac.uk/download; entry AF-Q8WZ42-F71-model_v4_14001–154,001 accessed 23rd Nov 2022); A77-A78 corresponds to the crystal structure with PDB code 3LPW; A84-A86 was elucidated in this study and A80-A82 was reconstructed in this study from homology models (obtained from the AlphaFold database as above) structurally aligned to the crystal structure of A84-A86

The conservation of linker sequences in titin A-band’s tandems allowed us to model the conformation of the A80-A82 tandem (3’-5’) in repeat C4, which could not be experimentally determined in this study (Fig. 6). Similar to A84-A86, A80-A82 contains domain pairs linked by the relative inflexible [N/Y]PF sequence followed by a [D]PI-type linker and, thus, it could be expected that its conformational dynamics closely resembles that of A84-A86. The availability of structural models for A77-A78 (Bucher et al. 2010), A80-A82 and A84-A86 in this work completes now a molecular understanding of poly-FnIII tandems of the representative C4 super-repeat.

### 3D-Mapping of sequence conservation patterns

Finally, we used structural models to assist the identification of putative binding interfaces on titin that might mediate the interaction with thick filament components. For this, we mapped conserved residues specific to each domain position within C-zone super-repeats onto the 3D-models available, aiming to reveal conserved surface clusters that might act as “hot spot” interaction loci in titin. We reasoned that if the super-repeat structure of titin’s A-band reflects the periodicity of the thick filament, then binding interfaces for the same filament proteins must be conserved positionally within the super-repeats. To reveal surface residues that could be involved in such interactions, we identified the differential sequence conservation specific to each FnIII-domain position (see Methods) in the super-repeats C2-C11, which are the more functionally homogeneous region of titin’s A-band. In brief, we applied a conservative approach, where sequences for all domains within an equivalent position in the super-repeat were grouped, and residues globally conserved across all FnIII (Supp Fig S1 & S5) as well as residues conserved in the same domain type at different super-repeat positions (Supp Fig S3) removed. Conservation in the remaining sequence was then analysed (Supp Fig S4 & S6). Our study revealed that for most domain positions within the super-repeat the conservation of surface residues is only small and dispersed, with merely a handful of residues conserved in each case (Fig. 6). This, with the exception of the first Ig domain (position 1 in the super-repeat) and the FnIII domain in position 5’ (position 7 in the super-repeat), which showed defined conserved sequence clusters on their surfaces (Fig. 6& Fig S8). Specifically, in the latter, 11 residues (out of 19 conserved in total) co-localized on the surface of the domain forming a conserved cluster centred around the sequence motif [LPDGR]xxxxx[FTN]xx[D/E] (Fig. 6, Fig S4 & S8). We speculate that this surface feature, and thus this super-repeat position, is of functional relevance to the scaffolding role of titin in the thick filament. Interestingly, FnIII domains in the C-terminal tandem 3”-5” did not present defined conservation areas on their surfaces. Thus, we propose here the two super-repeat positions above - Ig in position 1 and FnIII in position 7 - as important candidates in supporting interactions in the A-band. While the Ig has been shown to interact with MyBP-C (Freiburg and Gautel 1996), no role has been assigned to the domain in position 7 to this date. This domain and its conserved residues here identified are worth of experimental exploration.

## Discussion

**Fig. 7:**
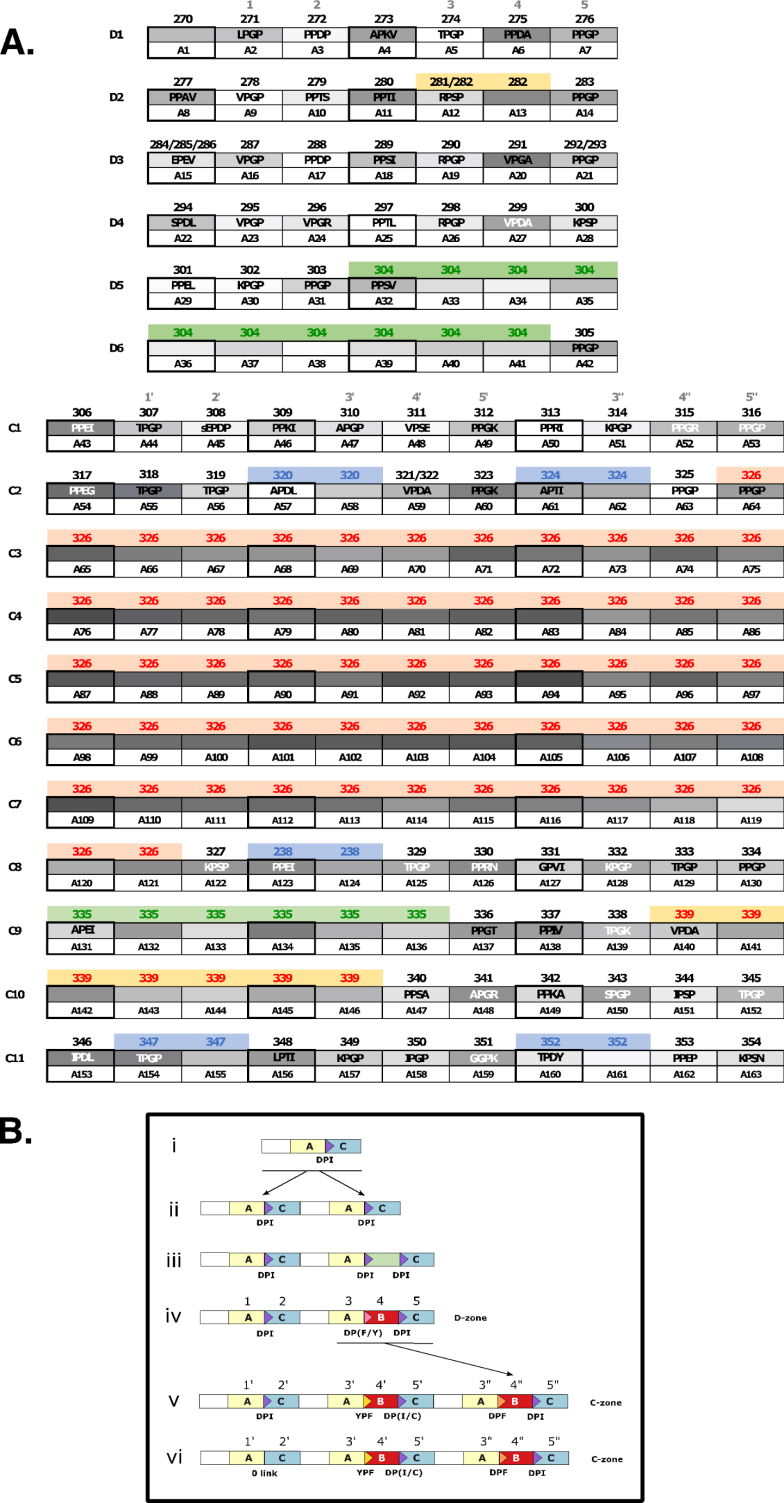
**Revised evolutionary model of titin’s A-band super-repeats**. **(A)** Exon structure of titin’s A-band. Exon numbers are written above the protein domains. Longer exons are coloured to ease visual identification. Protein domains are shadowed in grey to indicate conservation as in Fig. 2. Ig domains are indicated by a thicker box. The four protein residues at the beginning of each exon are given; **(B)** Fibronectin domains are labelled (A, B, C) and coloured-coded as in Fig. 1. Ig domains are shown as white, non-annotated boxes. Linker sequences are indicated as small triangles between FnIII domains and their consensus sequences given. In (i) to (iii), the DPI motif is proposed as the ancestor linker sequence

The large protein titin has emerged through serial genetic duplication events that are evidenced by its domain composition, where groups of domains repeat themselves along the chain forming super-repeats. Of these, super-repeats in the A-band region of titin are best conserved. We have revisited sequence conservation in this region of titin by performing a multi-dimensional analysis of pairwise sequence similarity in PaSiMap (Su et al. 2022) for domain components of the D- and C-zone repeats (Figs. 1 and 2). This revealed a clear gradient of conservation at the tandem repeat level, where the centre of the C-zone (repeats C4-C6) presents best defined systematic features followed by the flanking repeats (C3, C7), with conservation progressively decaying towards the N- and C-terminal repeats, the termini being the most diversified parts. High diversification is also encountered across the full D-zone. Given that C-zone repeats C2-C11 are thought to support similar interactions within the thick filament, this effect cannot be explained by differences in functional restraints during evolution. Instead, the degree of domain conservation likely reflects the evolutionary age of the components of the titin chain, with the central part of the C-zone having evolved later and, thereby, being evolutionarily more recent than the rest, suggesting that titin has become progressively extended into its middle section. In agreement with this deduction, the D-zone, with its shorter repeat unit that indicates it to be the older element, is the most highly diversified region in titin’s A-band with its domain components barely presenting systematic features (i.e. located close to the origin in PaSiMap vector maps; Fig. 2A). Interestingly, protein conservation in A-band repeats displays a certain correlation with exon structure (Fig. 7A). It can be observed that the most conserved, central part of the C-zone corresponds to a single long exon, while domains in the most divergent parts mostly correspond to individual or short exons. The potential influence of such exon structure in the past or future evolution of titin’s A-band is worth of reflection.

Notably, sequence conservation extends to interdomain linker sequences, which determine the relative arrangement of domains in the titin chain. Domain arraying dictates chain conformation and dynamics, knowledge which aids the evaluation of potential consequences of sequence polymorphisms and mutations in titin and, thereby, to clarify their linkage to disease. Residue positions at tight domain interfaces present less tolerance to change (Fleming et al. 2020) and can lead to disease through complex mechanisms (Bogomolovas et al. 2016). Linkers between Ig-FnIII and FnIII-Ig domains in A-band tandems cannot be analysed reliably at this time as 3D-structures of such domain pairs are not available. To date, 3D-structures of Ig-FnIII pairs from titin exist for M-line A169-A170 (Mrosek et al. 2007) and I110-I111 from the I/A-band junction (Stergiou et al. 2023), but the linkers in these pairs are not representative of those in titin’s A-band tandems. Thus, we focused here on the inspection of FnIII-FnIII linkers (Fig. 5). An analysis of intron/exon boundaries (Fig. 7A) shows that linker sequences are C-terminal components of FnIII domains, with which they are likely to have duplicated and co-evolved. This allows us to propose a revised model for the evolution of titin’s A-band (Fig. 7, i-vi) that expands earlier models (Labeit et al. 1992; Higgins et al. 1994; Kenny et al. 1999). In essence, the D-repeat formed first by duplication of a primary Ig-FnIII-FnIII tandem (i, ii), event that was then followed by the duplication of an FnIII domain accompanied by its C-terminal linker sequence (iii), which resulted in the Ig-FnIII-FnIII-FnIII repeat unit (iv). Given the close conservation of interdomain linkers between positions 1–2 and 4–5 (Fig. 5), it could be speculatively considered that the FnIII type B in the D-zone might have originated from a FnIII type A in position 1. Subsequently, the longer C-zone repeat must have formed by duplication of the last D-zone unit (v). Finally, the linker sequence between the first FnIII-FnIII pair must have been lost (vi), apparently within the first duplication event of the C-zone super-repeat. This is supported by the fact that the linker remains for the tandem 1’-2’ in C1, but not the subsequent repeats. This also suggests that repeat C1 is the ancestor of the C-zone super-repeats, with subsequent duplication events expanding titin progressively in its middle C-zone region as suggested by sequence conservation patterns (Fig. 2B). The evolutionary disappearance of the 1’-2’ linker early on in C-zone evolution might be of functional significance as linkers dictate inter-domain distances and, thereby, influence the position of anchoring sites on titin (Zacharchenko et al. 2015). In the current case, each three-residue linker in extended conformation would cause a shift in the titin chain of ~ 1 nm, amounting to a total length reduction of ~ 10 nm for the full C-zone (for comparison, this is equivalent to the removal of 2–3 FnIII domains). This could be expected to be of relevance for the templating role of titin in the thick filament.

In this study, we advance the structural understanding of titin’s A-band by elucidating the crystal structure of the triple FnIII-tandem A84-A86. The structure reveals two closely related extended conformations. This is in agreement with a previous SAXS study of A84-A86 that also revealed its conformation to be extended in solution (Bucher et al. 2010). In fact, extended conformations have been observed by SAXS for all poly-FnIII and poly-Ig segments from titin segments reported to date, including A77-A78 and A80-A82 (Bucher et al. 2010), A59-A60, A60-A62 and A67-A68 (Tskhovrebova et al. 2010), Z1Z2, I65-I70 and I91-I94 (Marino et al. 2005). In agreement with such observations in solution, all crystal structures of poly-domains from titin elucidated to date exhibit extended arrangements also in the crystalline lattice: namely, PDB entries 2NZI (Mrosek et al. 2007); 2ILL (Müller-Dieckmann et al., 2007); 2J8O (Müller et al. 2007); 3B43, 2RJM and 2RIK (von Castelmur et al. 2008); 3LPW (Bucher et al. 2010); 5JDE and 5JDD (Bogomolovas et al. 2016); 6FWX (Hill et al. 2019); 6SDB (Nesterenko et al. 2019); 7AHS (Stronczek et al. 2021); 8BXR and 8BVO (Stergiou et al. 2023); and 3LCY (unpublished). This is irrespective of whether the segments consist of poly-Ig, poly FnIII or have a mixed Ig-FnIII composition. It is also not influenced by the length and nature of the linker sequences. Thus, it can be concluded that extended conformations are common to recombinant, short segments of titin *in vitro*, independently of their composition. It could be rationalized that since domains within a tandem do not interact robustly with other domains up- or down-stream in the titin chain, bent conformations are not stabilized and, thereby, are short-lived and lowly abundant in the sample population. Consistent with this view, a single crystal structure exists that shows connected Ig-domains in a closed (“V” shape) conformation. This is the structure of Z1Z2 (PDB 2A38; Marino et al., 2006), where the closed conformation was stabilized by the binding of a Cd^2+^ ion (part of the crystallization mother liquor) between the Z1 and Z2 domains. It can be concluded that opening/tilting angles of extended domains arrangements in a titin tandem are typically small and not particularly informative, since not being specific to the tandem under study. On the other hand, domain torsion along the longitudinal molecular axis of the tandem is influenced by the packing of domains in the chain and is defined by linker sequences and the loop structure of individual domains (Zacharchenko et al. 2015), being characteristic of tandem type. As observed in other tandems from titin, also in A84-A86 individual domains adopt alternate torsional arrangements that minimize mutual steric hindrance along the chain (Fig. 4B).

Domain rotational arrangements in the titin chain are influenced by linker sequences, which also determine interdomain distances, contributing to the templating role of titin. Three main types of linker sequences are identified in the A-band: “zero-length” linkers, [N/Y]PF and [D]PI motifs. The previously available crystal structure of A77-A78 illustrates the tight domain interface generated by “zero-length” linkers that result in multiple domain-domain interactions and rigid tandem arrangements (Bucher et al. 2010). The structure of A84-A86 in this work illustrates the properties of [N/Y]PF and [D]PI motifs. These do not result in domain-domain interactions and are more flexible. Given the sequence conservation of linker motifs in titin’s A-band (Fig. 5) and that modern bioinformatics permits now the accurate prediction of 3D-structure at the individual domain level, the characterization of linker conformations can enable the modelling of a large fraction of titin’s A-band. The availability of 3D-structures can assist the interpretation of positional sequence conservation patterns within domains in C-zone super-repeats. Our analysis of such conservation (Fig. 6 & Fig S8) revealed that very few residues are positionally conserved within each domain in the repeat, that those conserved only form surface clusters in non-consecutive domains in positions 1 and 7 of the super-repeat and that conservation at domain-domain interfaces is limited to linker sequences, which might also participate in binding to thick filament components. Our conservation analysis complements a recent study (Tonino et al. 2019) that focused on domains at the interface between super-repeats D6-C1 and C1-C2, which are known not to bind MyBP-C. That analysis was restricted to the first and last domain positions within the super-repeats (i.e. positions 1–3 and 8–11). It compared the mentioned repeat interfaces to those in the rest of the C-zone in order to identify residues with the potential to hinder MyBP-C binding. The binder residues proposed from our current analysis and those previously suggested by Tonino et al. to conflict with binding constitute a complementary set to experimentally investigate the role of titin in the structural organization of thick filament components.

### Electronic supplementary material

Below is the link to the electronic supplementary material.

Supplementary Material 1

Supplementary Material 2

Supplementary Material 3

## Data Availability

Model coordinates have been deposited with the Protein Data Bank under accession code 8BNQ, and diffraction data are deposited at 10.5281/zenodo.7428766.
