# Supplementary material for: Molecular insights into titin’s A-band
Source: J Muscle Res Cell Motil. 2023 May 31;44(4):255–70. doi: 10.1007/s10974-023-09649-1 (PMC10665226; doi:10.1007/s10974-023-09649-1)

**SUPPLEMENTARY MATERIAL**

**Molecular insights into titin’s A-band**

Jennifer R. Fleming^1^, Iljas Müller^1&^, Thomas Zacharchenko^2¶&^, Kay Diederichs^1^, Olga Mayans^1^

*^1^ Department of Biology, University of Konstanz, 78457 Konstanz, Germany; ^2^ Institute of Integrative Biology, University of Liverpool, Liverpool, L69 7ZB, UK.*

**Fig S1: Global MSA alignment of FnIII domains from titin’s A-band showing consensus residues.** For each position, the SoP (sum-of-pairs) score is given, where the higher the score the greater the consensus for that alignment position. SoP scores >0.5 (see FigS2) and were taken to constitute the global conservation consensus for A-band FnIII domains and are marked in pale blue.


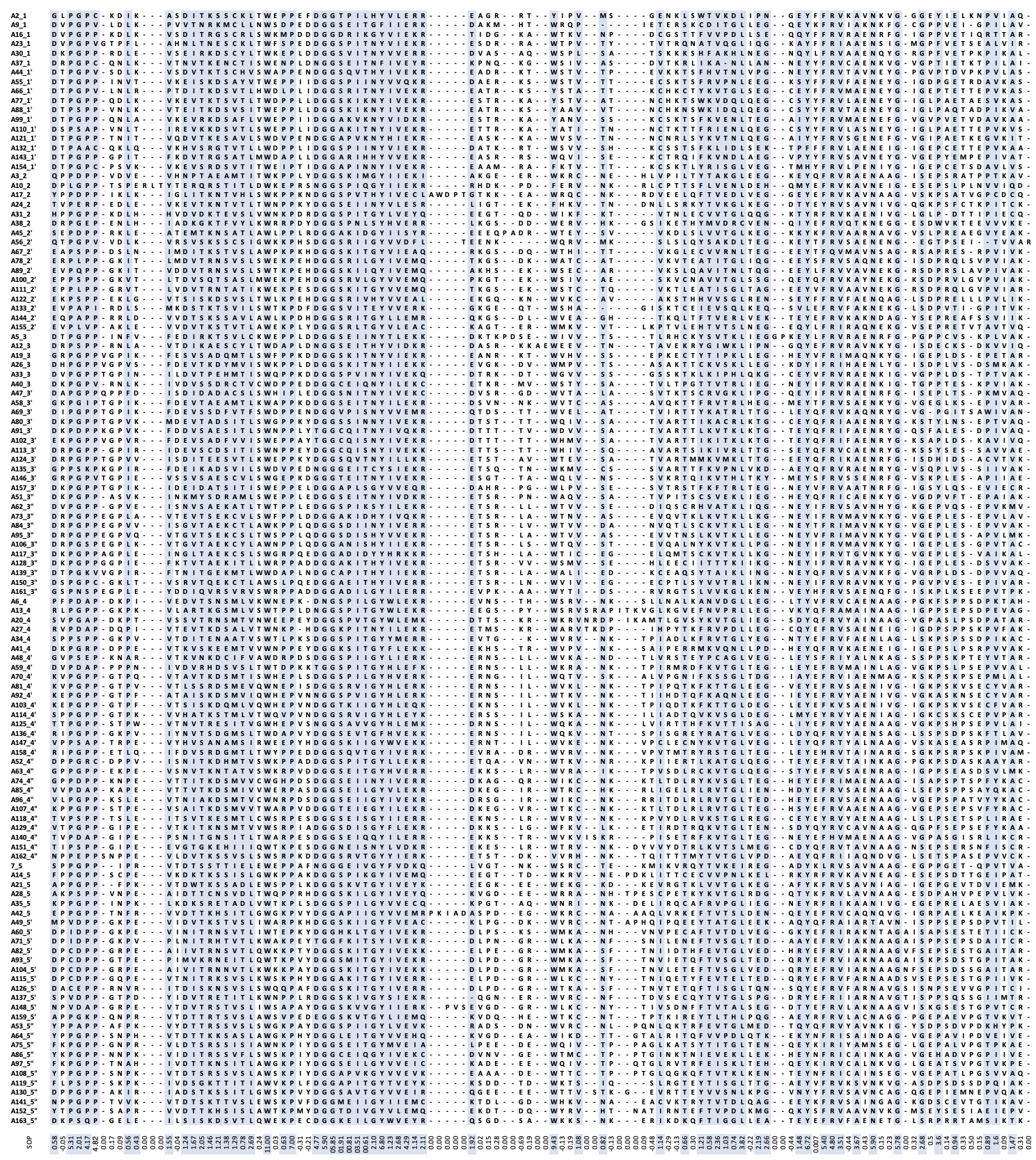


**Fig S2: RMSD-based calculation of the global consensus in sequence conservation in A-band FnIII domains**

**A.** RMSD values from the comparison of two PaSiMap vector maps. Blue line; RMSD values calculated between the PaSiMap output for the original, unedited MSA (native MSA) and the PaSiMap output for each derivative MSAs, where positions had been cumulatively removed in order of decreasing conservation. Red line: RMSD values calculated between the PaSiMap output for the original, unedited MSA (native MSA) and a decoy derivative MSA, where positions had been cumulatively removed at random. Here, the RMSD value shown is an average of three decoy derivative MSA generated at random; **B.** For each cumulative sequence removal, the difference between both RMSD values shown in A. (blue, red lines) is displayed. A horizontal or decreasing slope in the weighted ΔRMSD (see Methods) curve is the point where removing a position by conservation is no longer different to removing a residue at random. This point is marked with a vertical line and corresponds to a SoP score of 0.5.

**
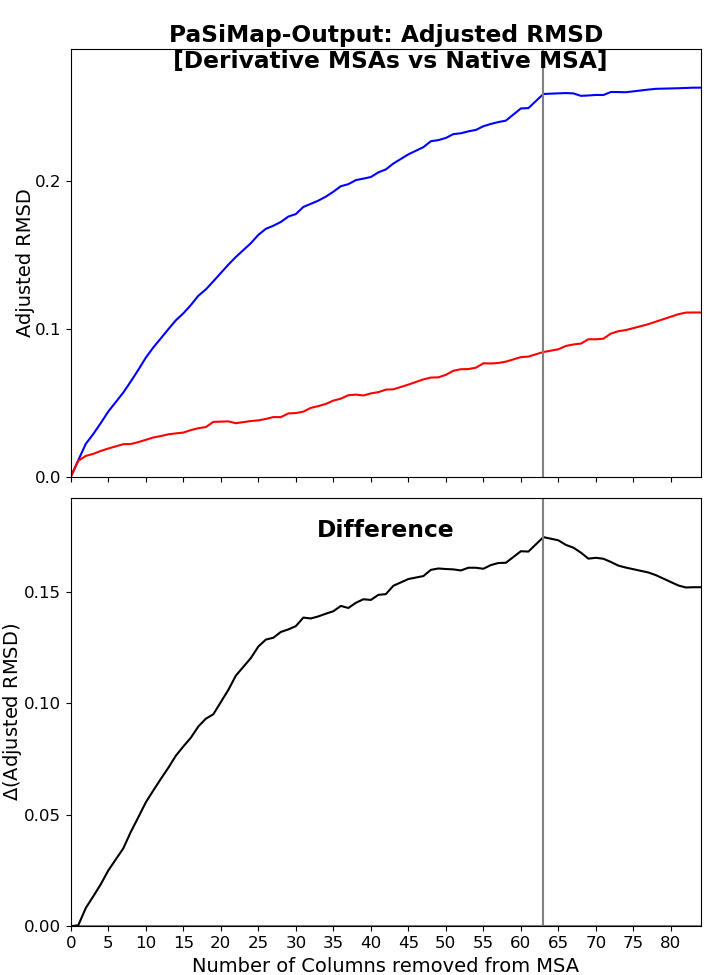
**

**Fig S3: Group MSA alignments of FnIII domains from titin’s A-band showing consensus residues.**

Positions in grey correspond to SoP scores >0.5 and were taken to constitute the global conservation consensus for A-band FnIII domains. FnIII-type specific residues are considered as those that match the criteria of a SoP > 1 and over 90% occupancy and are shown in yellow.


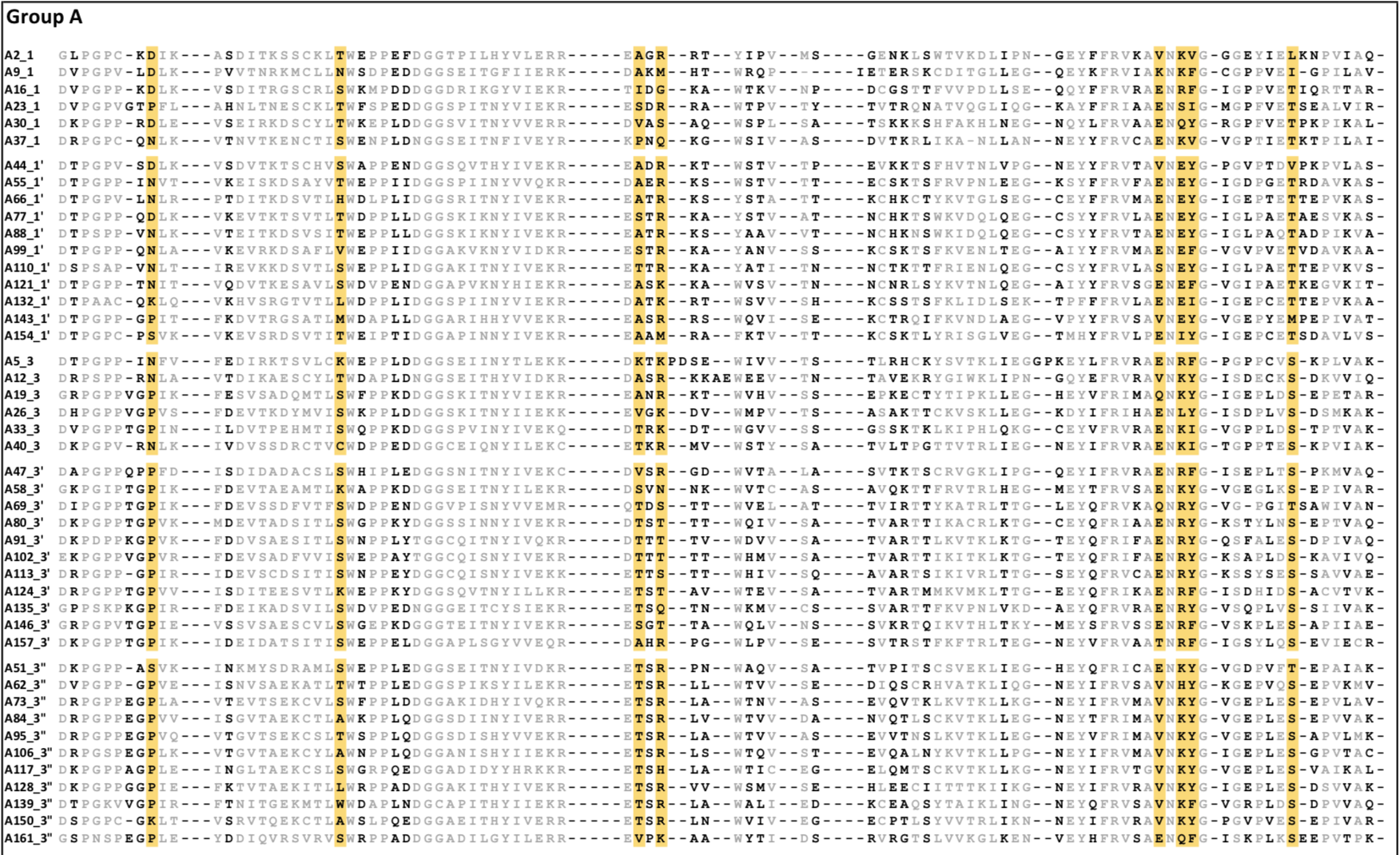


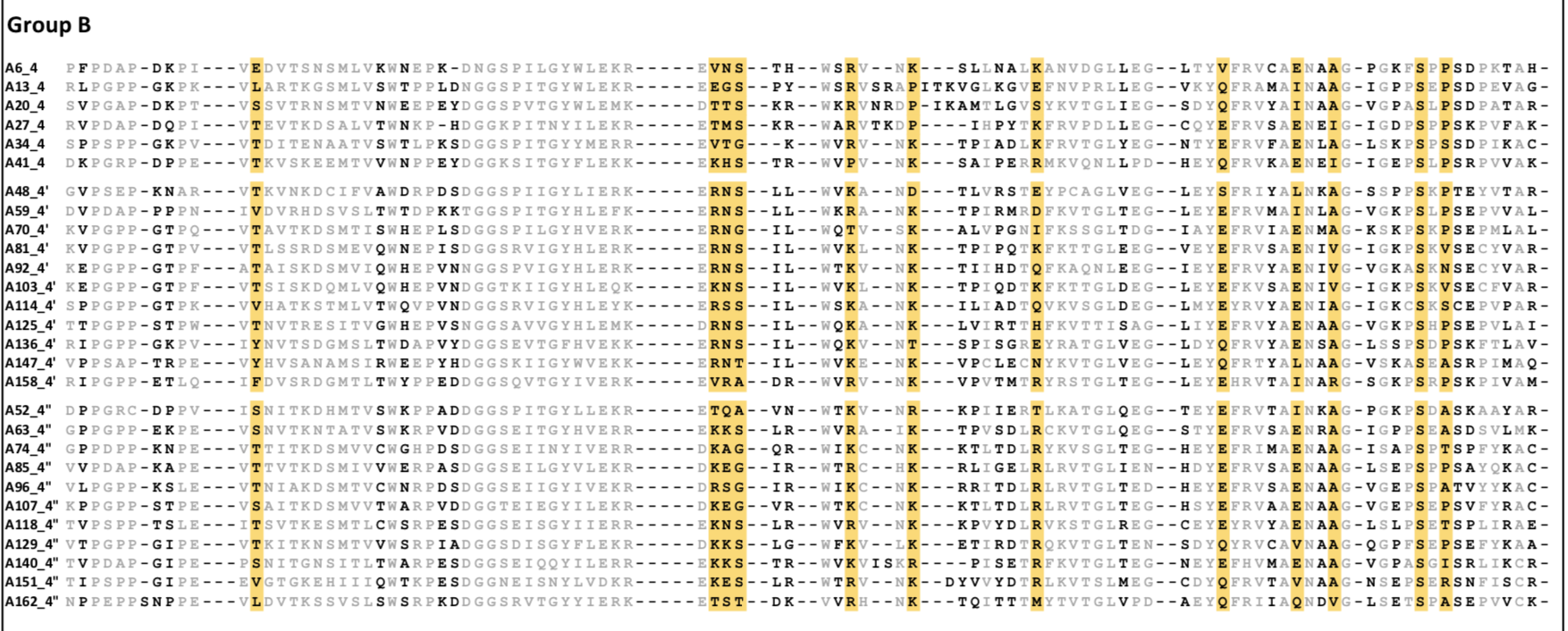


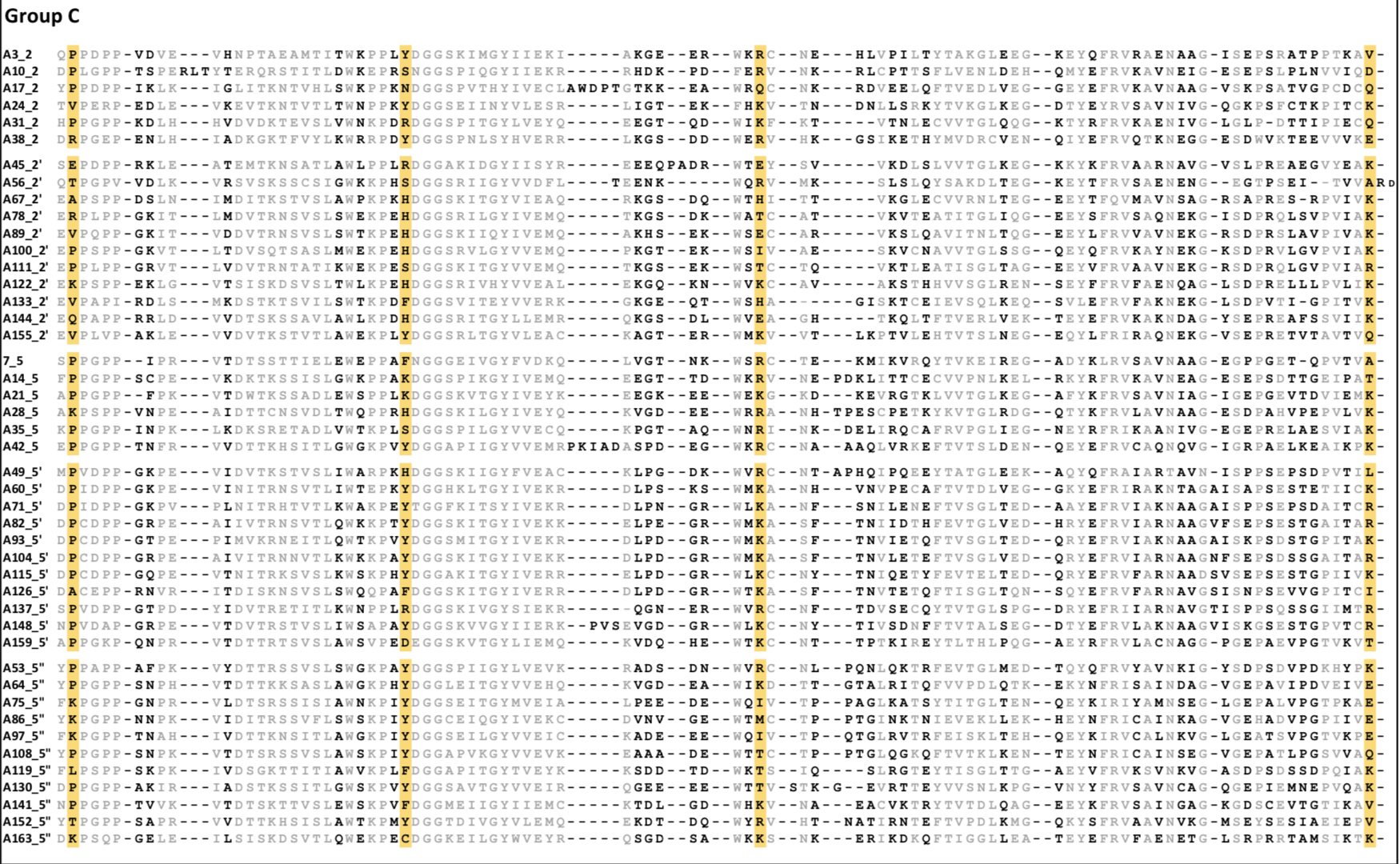


**Fig S4: Sequence consensus for domain position within the C-zone super-repeat.**

Residues conserved globally and within the FnIII-type plus those also conserved within each subgroup are shown in grey. Residues specific to each domain position within the super-repeat (selected according to the criteria of a SoP > 1, at least 90% occupancy of the position and at least 70% sequence identity) are shown in orange.

**Type A subgroups**

**
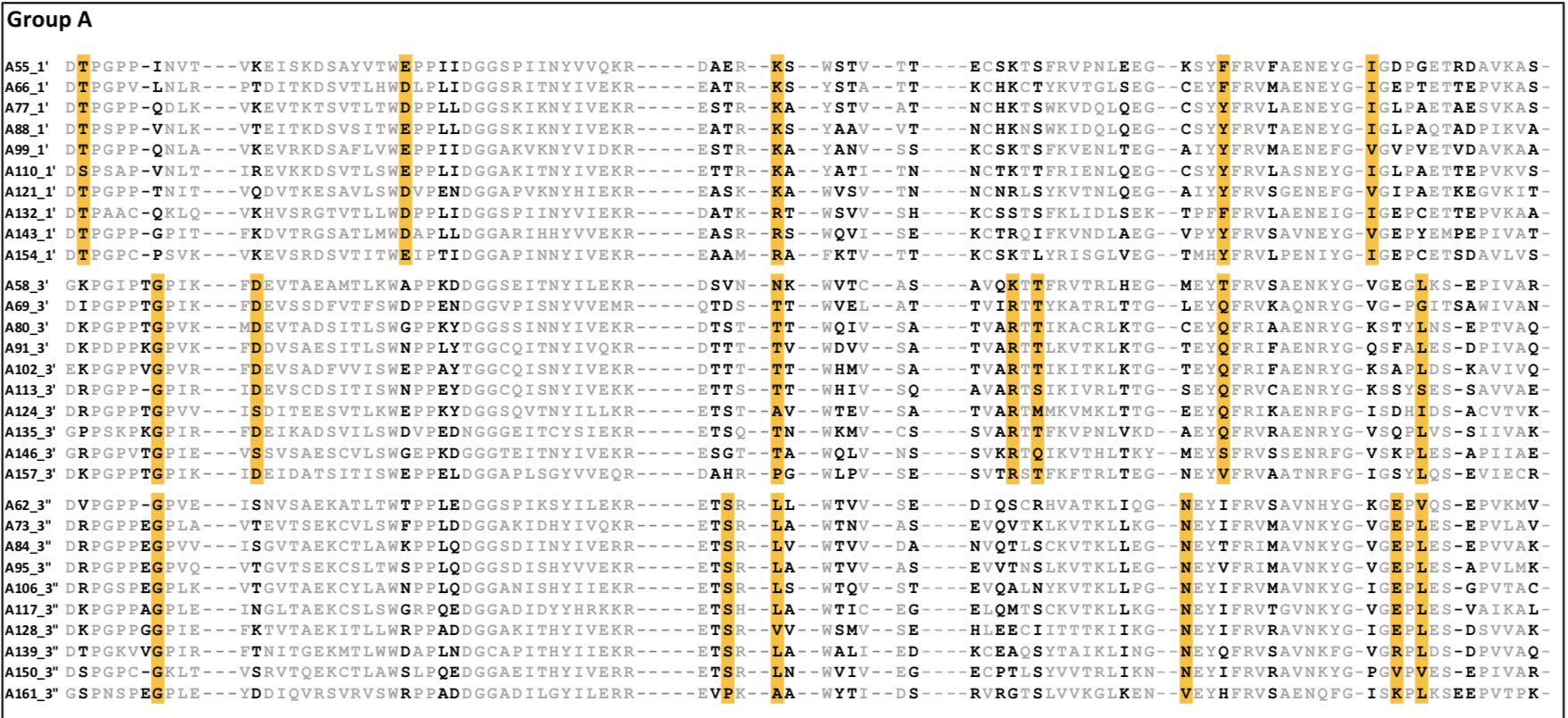
**

**Type B Subgroups**


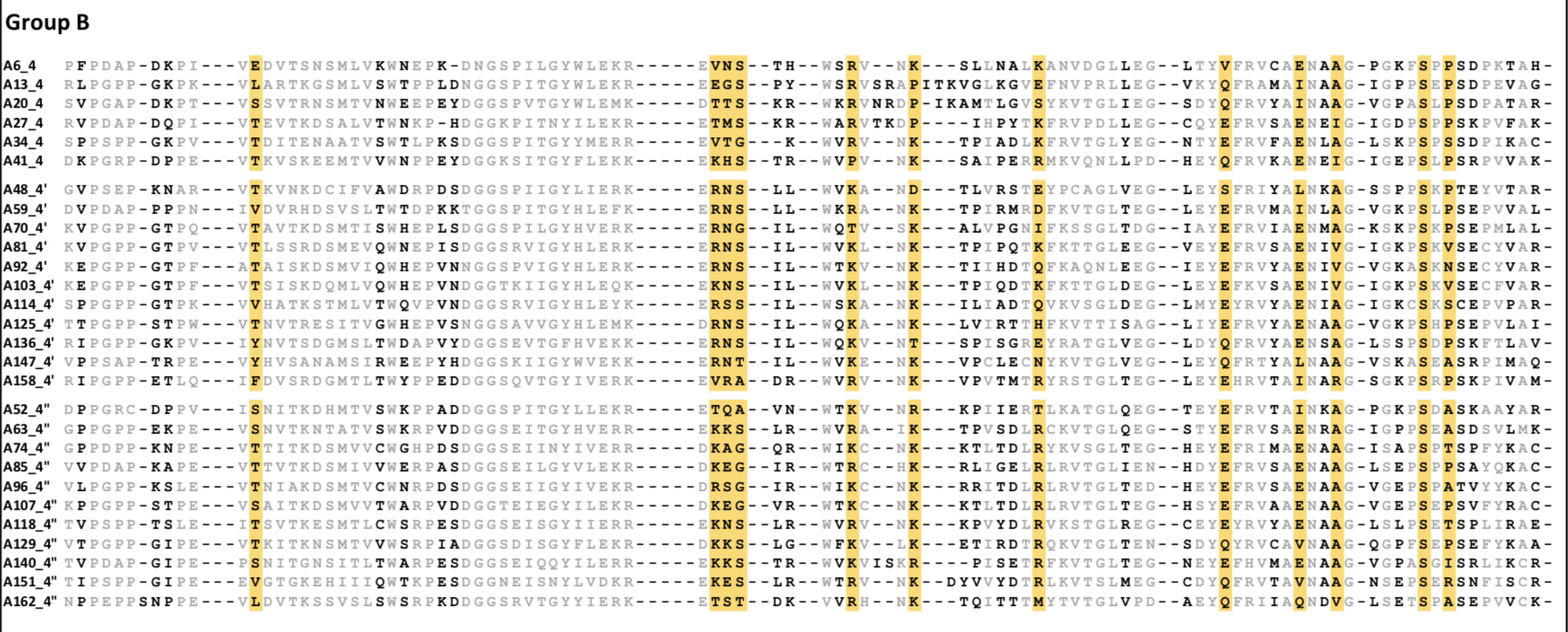


**Type C subgroups**


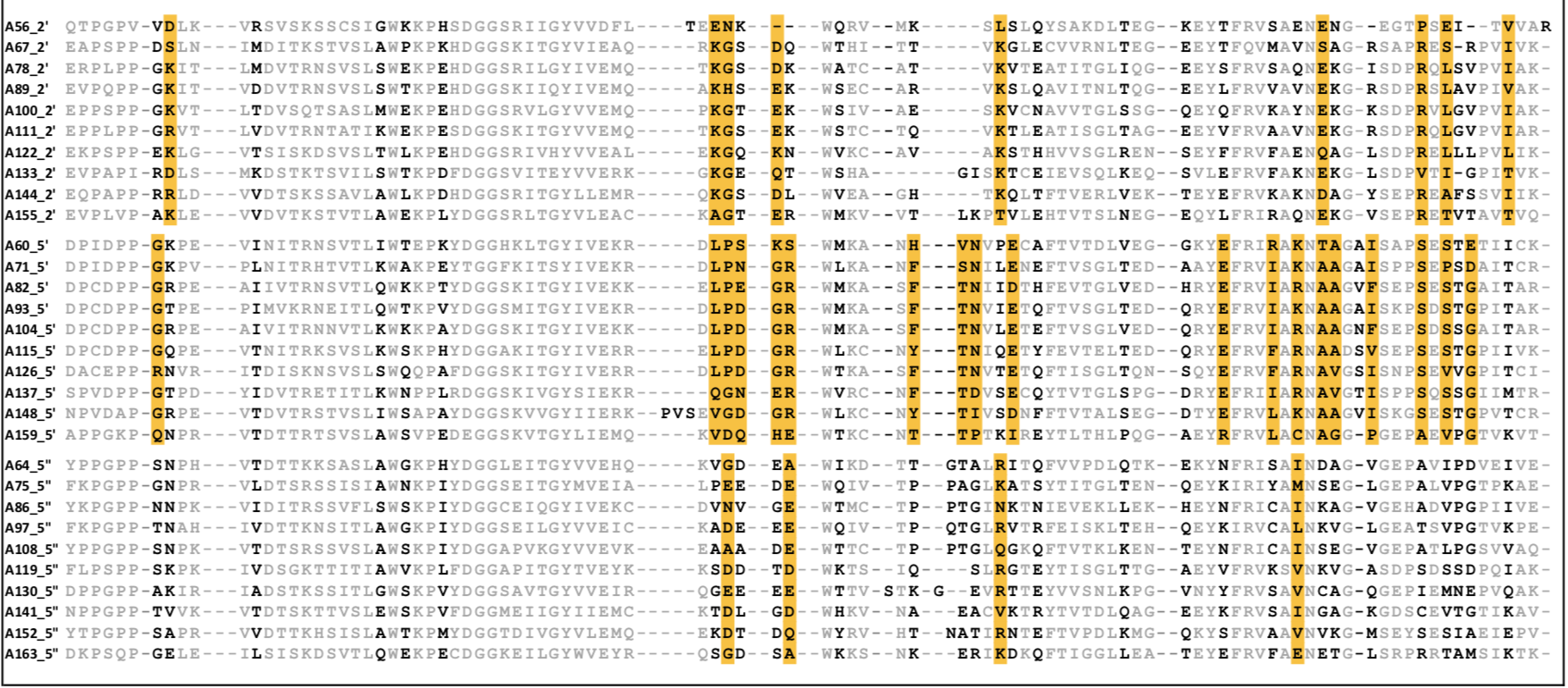


**Fig S5. Global MSA alignment of A-band Ig (and I105) domains showing consensus residues.** For each position, the SoP (sum-of-pairs) score is given, where the higher the score the greater the consensus for that alignment position. SoP scores >0.5 and were taken to constitute the global conservation consensus for A-band Igs domains and are marked in pale blue.

**
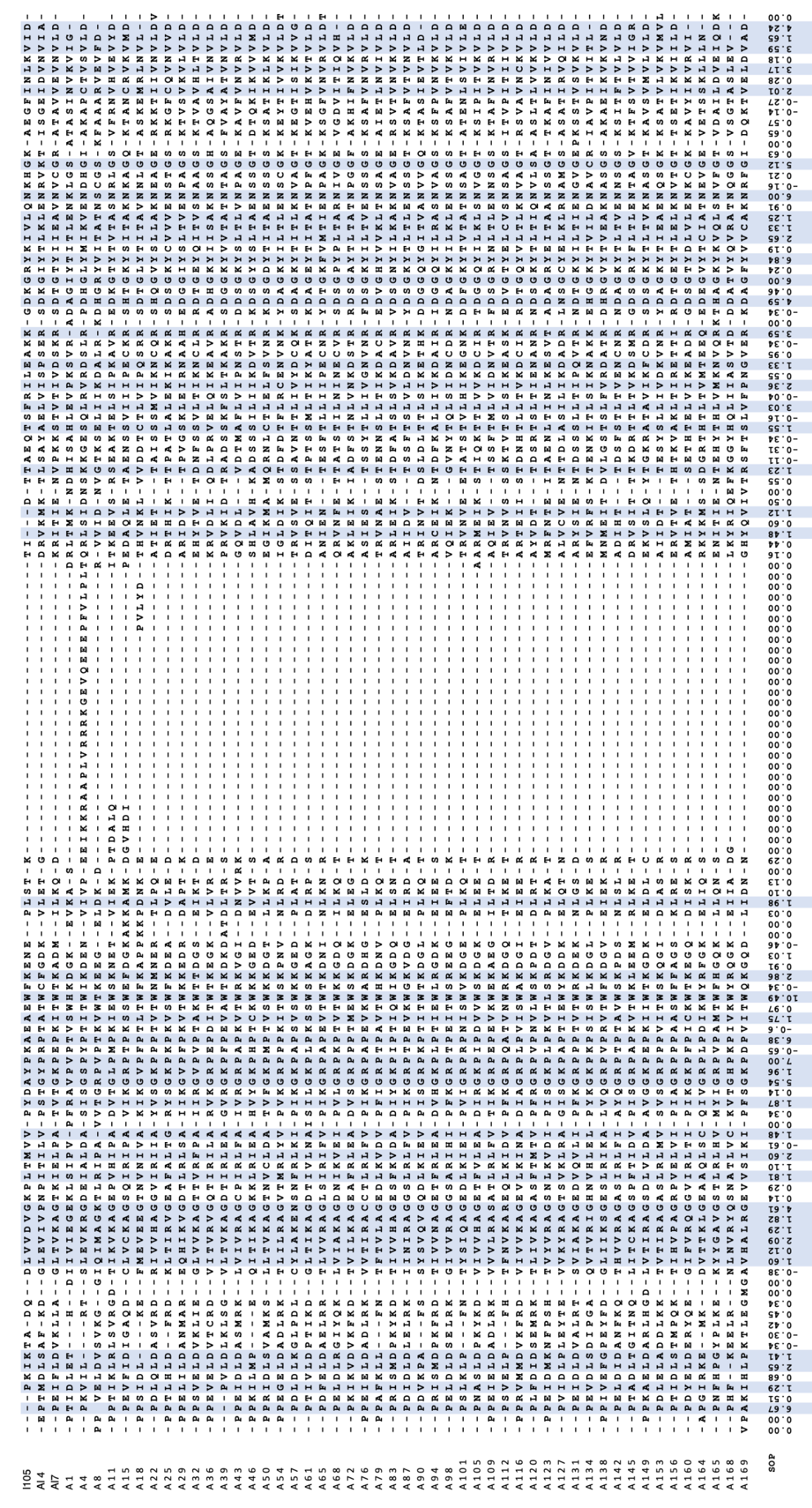
**

**Fig S6: Sequence consensus for Ig domain position within the C-zone super-repeat.**

Residues conserved globally and within the A-band Ig are shown in grey. Residues specific to each domain position within the super-repeat (selected according to the criteria of a SoP > 1, at least 90% occupancy of the position and at least 70% sequence identity) are shown in orange.

Position i (Ig1)


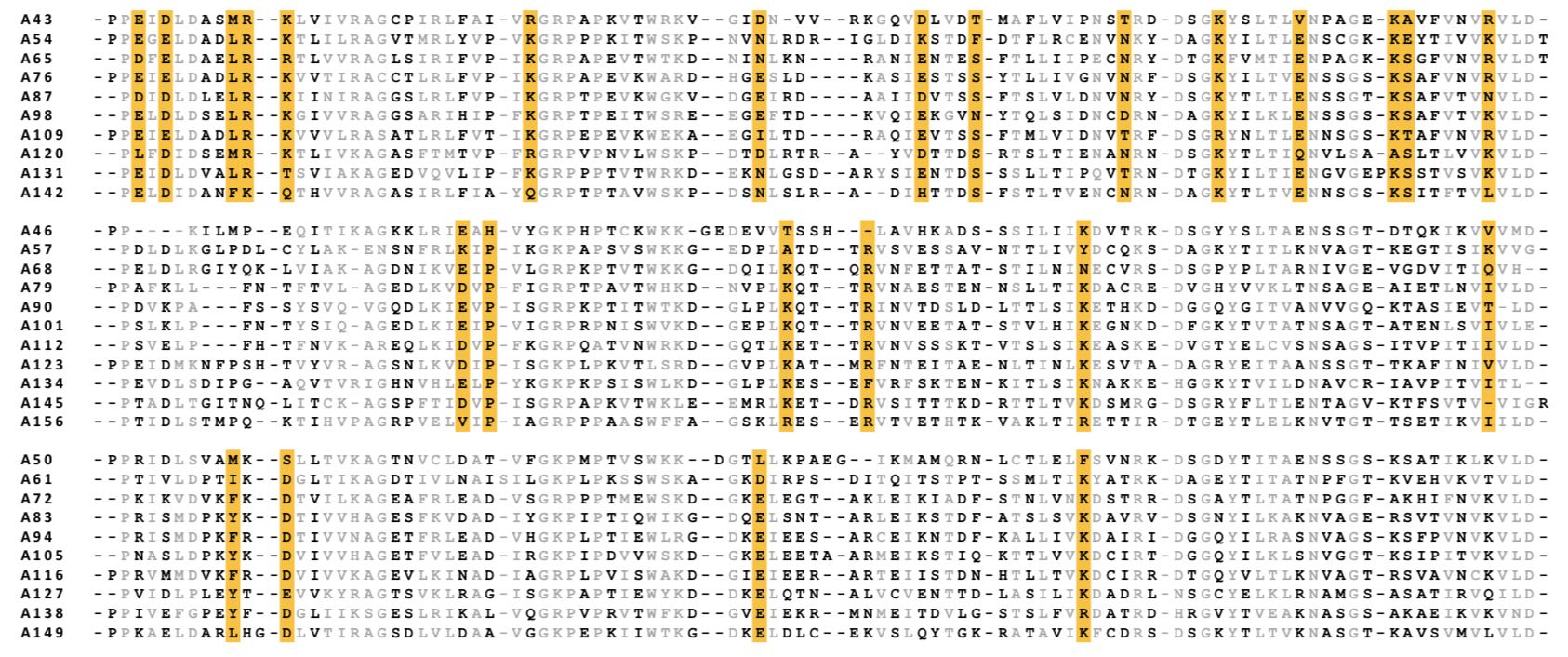


Position ii (Ig2)


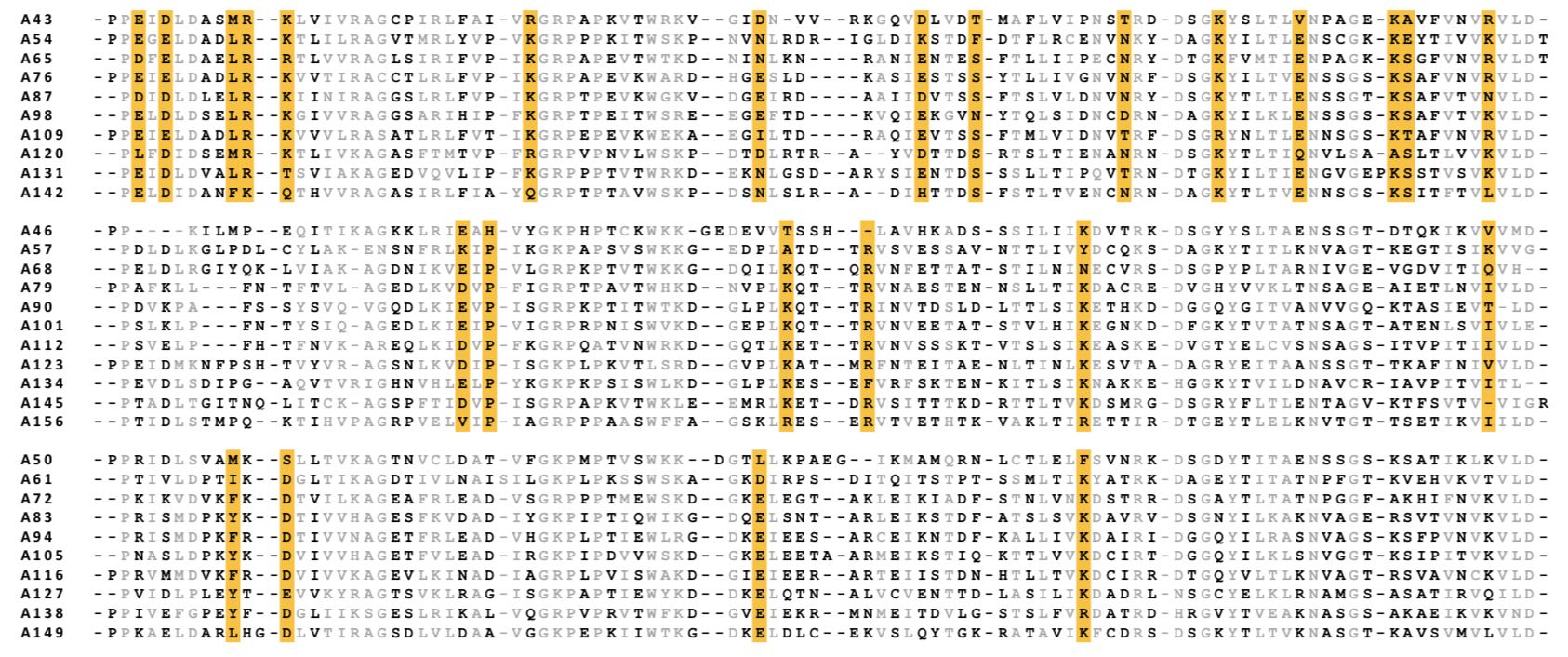


Position iii (Ig3)


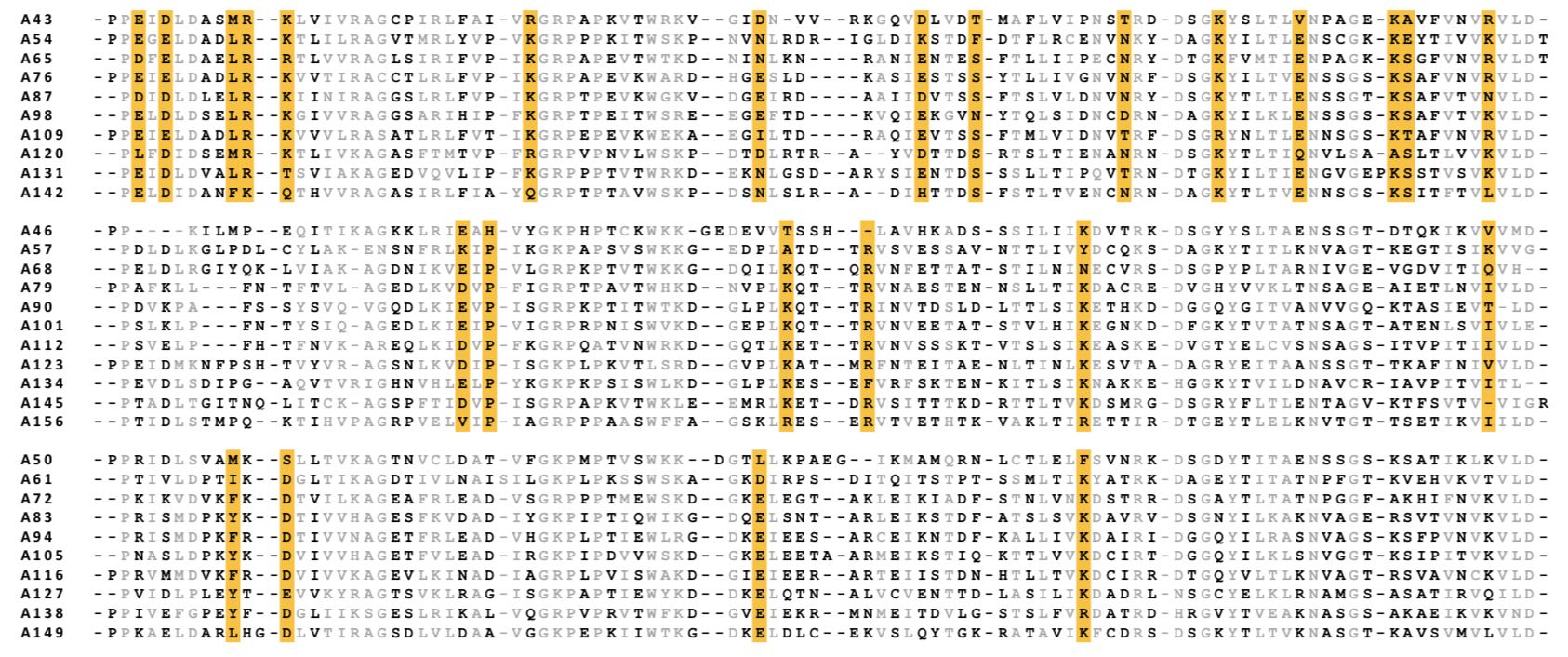


**Fig S7: Domain interfaces in the extended conformation of the remaining copy of A84-A86 in the crystal structure**

**A84-A85 A85-A86**


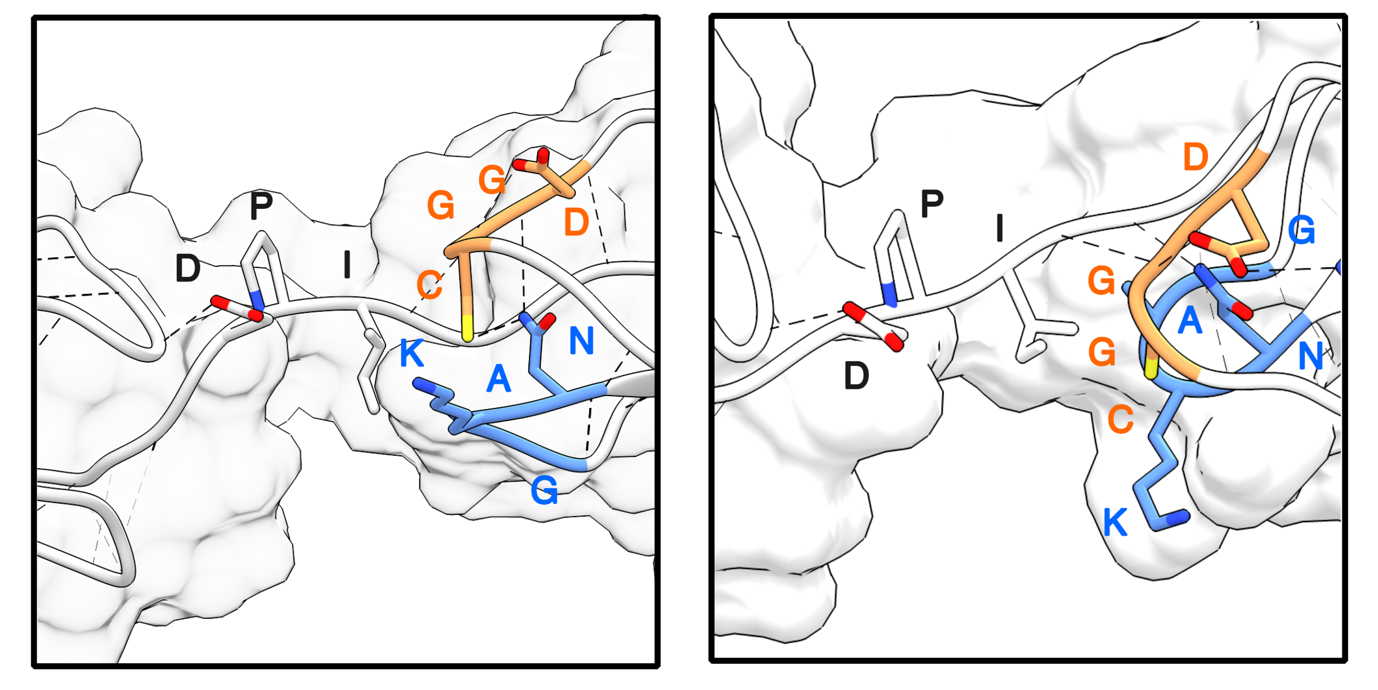


**Fig S8: Residue composition of patches of positionally conserved surface residues**

1. Conservation of residues in the first Ig of super-repeats C2-C11 displayed for the domain in C4; **B.** As A. but for FnIII in position 7 of the super-repeat displayed on domain A82.


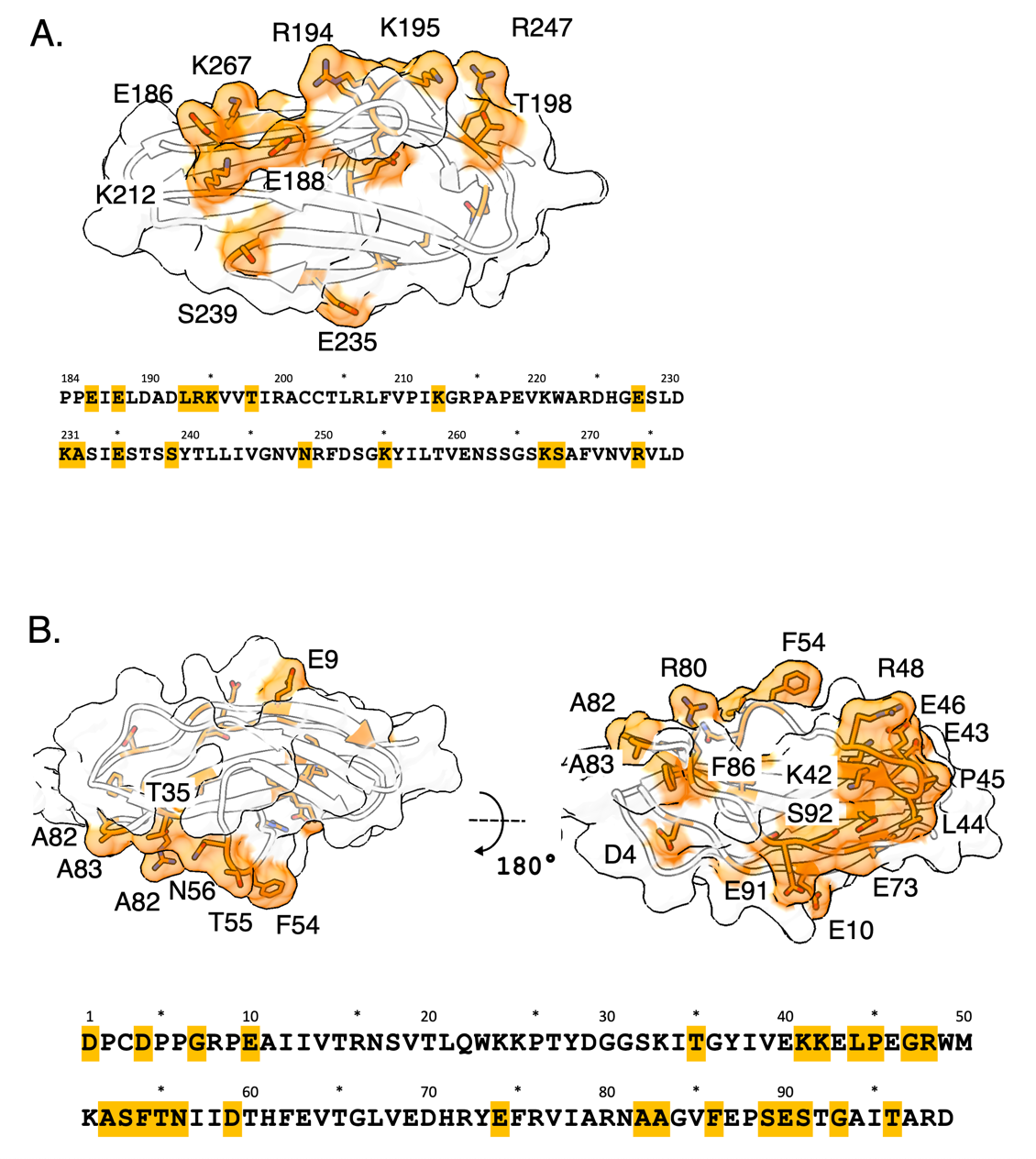

Supplement: Supplementary file 2 — Supplementary Material 2 [file 10974_2023_9649_MOESM2_ESM.docx]
